# TMTP1-modified polymeric micelles for the inhibition of ovarian cancer metastasis and recurrence through enhanced photothermal-immunotherapy

**DOI:** 10.1016/j.mtbio.2025.101825

**Published:** 2025-05-04

**Authors:** Ling Wang, Jie Li, Danya Zhang, Songwei Tan, Guiying Jiang, Xueqian Wang, Fei Li, Ying Zhou, Pingbo Chen, Rui Wei, Ling Xi

**Affiliations:** a Department of Obstetrics and Gynecology, National Clinical Research Center for Obstetrics and Gynecology, Tongji Hospital, Tongji Medical College, Huazhong University of Science and Technology, Wuhan, Hubei Province, China; b Key Laboratory of Cancer Invasion and Metastasis (Ministry of Education), Hubei Key Laboratory of Tumor Invasion and Metastasis, Tongji Hospital, Tongji Medical College, Huazhong University of Science and Technology, Wuhan, Hubei Province, China; c Department of Obstetrics and Gynecology, The First Affiliated Hospital of Anhui Medical University, Hefei, Anhui Province, China; d Tongji School of Pharmacy, Tongji Medical College, Huazhong University of Science and Technology, Wuhan, Hubei Province, China; e Department of Gynecology, West China Second University Hospital, Chengdu Province, China

**Keywords:** Tumor vaccine *in situ*, Photothermal-immunotherapy, Cancer vaccine, Primary and metastatic tumor, Ovarian cancer

## Abstract

Metastasis and recurrence are the main causes of failure in the treatment of ovarian cancer (OC). Immunotherapy has brought new opportunities for cancer treatment, but the presence of the immunosuppressive tumor microenvironment limits its application in OC. Here, we prepared a kind of intelligent nanomicelles that could inhibit OC metastasis and recurrence by combining photothermal tumor ablation and immune remodeling. In this study, Indocyanine green (ICG), a photosensitizer and Monophosphoryl lipid A (MPLA), a Toll-like receptor 4 agonist, were encapsulated into the core of PEG-PLGA nanocarrier, and the surface was further modified with tumor-targeting peptide TMTP1. The formed TP1-IM nanomicelles with enhanced tumor accumulation could enable robust photothermal ablation of the primary tumor, and induce immunogenic cell death, release tumor-associated antigens, and damage-associated molecular patterns to form an endogenous *in situ* tumor vaccine by a single intravenous injection, which could effectively inhibit the growth of OC. Moreover, PTT with TP1-IM nanomicelles in combination with programmed cell death ligand 1 (PD-L1) checkpoint blockade could induce strong anti-tumor immune responses, relieve the immunosuppressive tumor microenvironment, and thus effectively inhibit the metastasis of OC. In addition, the combination strategy could produce long-term immune memory effects in treated mice to prevent the recurrence of tumors. Our work provides a new and individualized strategy for the treatment of OC and is expected for clinical transformation in the future for most components are clinically approved.

## Graphical abstract

**Figure:**
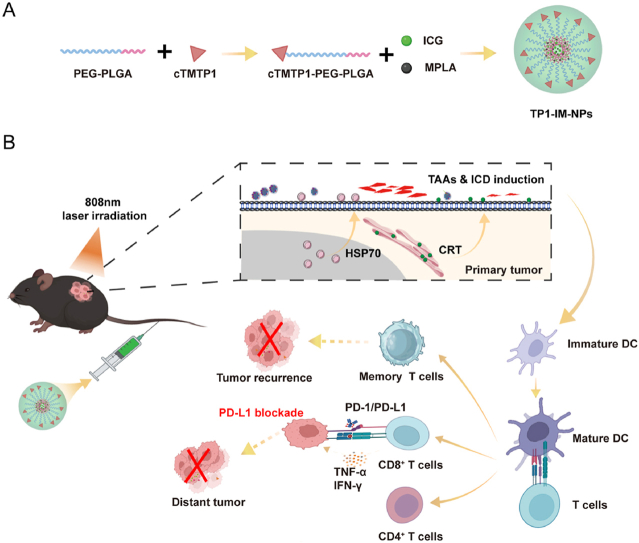

## 1 Introduction

Currently, ovarian cancer (OC) remains the gynecological malignant tumor with the highest mortality rate. According to the global cancer statistics, there were approximately 313,959 new cases of OC and 207,252 deaths in 2020 [1]. Due to a lack of early and effective screening methods, most OC patients diagnosed are already in the late stages, and the five-year survival rate is less than 30 %. Metastasis and the high recurrence rates have led to the poor prognosis of OC [2,3]. The current front-line standard for OC remains surgery and platinum-based chemotherapy. Although 80 % of patients initially respond to the treatment, most of them will relapse within one to two years and develop chemotherapy resistance [4]. In recent years, some new treatment methods have been proposed, such as anti-angiogenic inhibitors and poly ADP-ribose polymerase (PARP) inhibitors, but none significantly improved the overall survival rate of OC patients [[5], [6], [7]]. Therefore, to improve the prognosis of OC patients, it is urgent to develop a new OC treatment strategy that could not only eliminate primary tumor lesions but also track and kill residual tumor cells to inhibit the metastasis and recurrence of OC.

Immunotherapy is considered as a huge breakthrough in the field of cancer treatment by stimulating the body's immune system to attack solid tumors and circulating tumor cells. OC patients are expected to benefit from immunotherapy for OC is considered an “immunogenic tumor”, and anti-tumor immune responses could be detected in tumor tissues, peripheral blood, and ascites of OC patients. Several immunotherapy strategies, such as immune checkpoint inhibitors (ICI), adoptive T-cell therapies and tumor vaccines, have obtained effective clinical responses in several types of tumors [[8], [9], [10]]. Especially, tumor vaccines have many unique advantages by loading with tumor antigens to stimulate the body to produce antigen-specific immune responses. It can also produce long-term immune memory effects and inhibit tumor recurrence [[11], [12], [13]]. Tumor vaccines can be mainly divided into tumor-specific antigens-based vaccines and whole-cell cancer vaccines. Tumor-specific antigen vaccines can reduce the risk of autoimmunity and are expected to develop personalized vaccines with “cold tumors” [14]. However, the occurrence and development of tumors is a dynamic process characterized by an unstable genome. Therefore, it is difficult to determine the public and effective tumor new antigen, thus limiting the clinical application of tumor new antigen vaccines. Whole-cell cancer vaccines utilize autologous or allogeneic tumor tissue lysates that contain multiple tumor antigens, which are theoretically suitable for various solid tumors [[15], [16], [17]]. However, the complex processing process and uncertainty in preparing whole-cell cancer vaccines have led to unsatisfactory clinical trial results [18]. Therefore, it is necessary to explore an immunotherapy strategy that is easy to operate and has high specificity and efficacy.

To overcome this dilemma, some researchers have proposed the strategy of *in situ* tumor vaccines recently [19,20]. *In situ* tumor vaccines utilize tumor antigens released by necrotic or apoptotic tumor cells at the tumor site to produce an endogenous vaccine-like effect *in vivo* [[21], [22], [23]]. To stimulate a robust anti-tumor immune response, an ideal *in situ* tumor vaccine should induce immunogenic cell death (ICD) of tumor cells, release tumor-associated antigens, stimulate antigen-presenting cells to capture antigens, and activate T cells. Photothermal therapy (PTT), as a novel tumor treatment, utilizes photosensitizers to convert light energy into heat energy under laser irradiation, generating local hyperthermia to kill tumor cells. As a non-invasive, spatially controllable modality, PTT offers the advantage of selectively ablating tumor tissues with minimal damage to surrounding healthy structures and localized therapeutic action reduces the risk of systemic adverse effects, providing a safer and more targeted approach to ovarian cancer management [24]. In addition to directly destroying the primary tumor cells, PTT could also induce ICD to stimulate the body to produce antigen-specific anti-tumor immune responses, inducing an endogenous tumor vaccine effect. Compared to traditional radiotherapy or chemotherapy, PTT presents obvious advantages such as increased drug permeability in the tumor due to local hyperthermia, high spatiotemporal selectivity, minimal side effects, and simultaneous tumor imaging [[25], [26], [27], [28], [29]]. Clinical transformation is expected to be achieved by using an implanted NIR light source. Recent studies have reported that clinical devices based on endoscopes with embedded laser fibers could apply PTT to tumors deep inside the body. Kinoshita et al. developed a new type of parallel ultra-small composite fiberscope with a diameter of only 0.97 mm, which could simultaneously achieve optical imaging and photothermal therapy of peripheral lung cancer [30]. Singh et al. combined fluorescent endoscopes with nanomedicines to achieve fluorescence imaging-mediated photothermal therapy of gastrointestinal tumors [31]. Therefore, it is promising to achieve clinical transformation in ovarian cancer through invasive PTT in the future.

Photothermal immunotherapy, integrating PTT and immune modulation, has emerged as a promising combined approach for tumor treatment. PTT combined with immune adjuvants could induce a more potent vaccine-like effect *in situ* [[32], [33], [34]]. The spiky gold nanoparticles that Jiang et al. developed demonstrate excellent *in vivo* therapeutic effects in the breast mouse model [30], and the manganese-boosted NIR photo-immunotherapy strategy that Zhang et al. proposed shows great therapeutic efficacy against both primary and lung metastatic melanoma tumor growth [31]. Chen et al. showed that combining photothermal material polydopamine with immune adjuvant CpG oligodeoxynucleotide could effectively inhibit melanoma growth [35]. Further, the ICI combination will contribute to preventing tumor metastasis and recurrence by remodeling the inhibitory tumor microenvironment. Nanoplatform provides a useful strategy for simultaneously delivering multiple drugs with tumor specificity. Polymer micelles are stable colloidal solutions formed by self-assembling amphiphilic block copolymers in water. These micelles have a core-shell structure and could be used for solubilization and encapsulation of drugs. Hydrophobic drugs are encapsulated in the hydrophobic core of the micelles through hydrophobic interactions, while hydrophilic drugs could be attached to the surface of the material through physical interactions or chemical linkages [36]. Among various copolymers, polyethylene glycol-poly lactic acid-co-glycolic acid (PEG-PLGA) copolymer has distinct advantages such as good biodegradability, biocompatibility, and easy surface modification [37]. PEG-PLGA has been approved by the FDA for parenteral drug delivery systems. Moreover, active tumor targeting is critically important for improving the treatment outcomes of drugs and reducing systemic toxicity. Tumor-targeting peptide TMTP1 was screened by our team through the bacterial flagellar peptide library display system FliTrx. The amino acid sequence of TMTP1 is NVVRQ, which can target highly metastatic tumor cells [38]. Multiple studies have shown that TMTP1 could target various tumors such as prostate cancer, breast cancer, cervical cancer, osteosarcoma, and ovarian cancer [[38], [39], [40], [41], [42]], which demonstrated the potential for achieving active targeting of nanodrugs to ovarian cancer by modifying TMTP1 on nanocarriers.

**Scheme 1:**
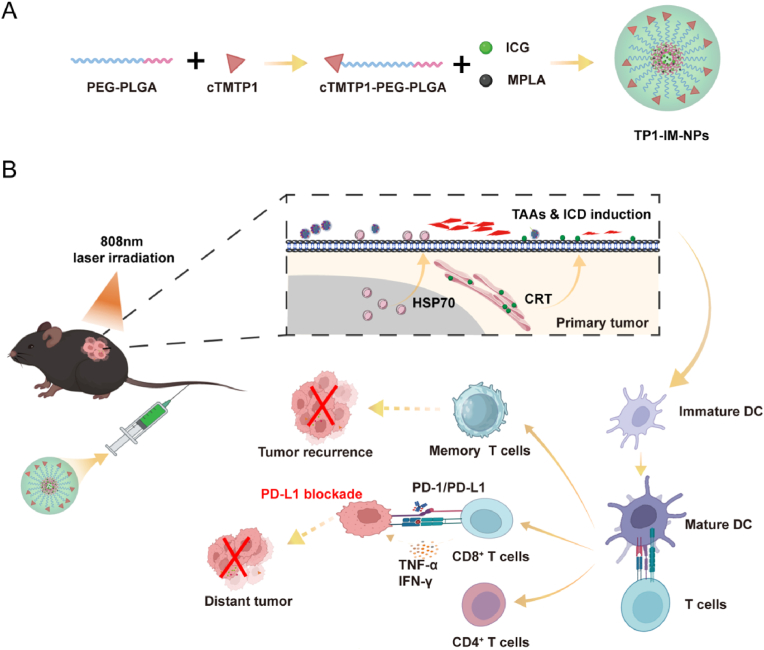
Schematic illustrations for the preparation (A) and anti-tumor effects of TP1-IM micelles by combining photothermal ablation and immunotherapy (B).

In this study, the near-infrared (NIR) fluorescent dye Indocyanine green (ICG) and immune adjuvant Monophosphoryl lipid A (MPLA, a Toll-like receptor 4 agonist) were selected for *in situ* eradication and vaccination of OC. Then, the tumor-targeted peptide TMTP1 was modified on the surface of PEG-PLGA nano-carriers to form TP1-IM micelles (Scheme1A). After a single intravenous injection, the accumulation of TP1-IM micelles in OC could be observed in real-time by fluorescent imaging. When the accumulation of drugs reached its peak, PTT-driven could ablate the primary tumor cells by controlled NIR laser irradiation. Afterward, apoptotic or necrotic tumor cells release tumor-associated antigens (TAAs) and induce ICD to activate dendritic cells (DCs) and initiate the body's adaptive immune response, inducing an endogenous tumor vaccine effect. Furthermore, by combining it with programmed cell death ligand 1 (PD-L1) checkpoint blockade, it could achieve tumor regression and rechallenge protection (Scheme 1B). Overall, our study provided a novel combinational strategy for OC treatment by enhanced photothermal therapy and immunotherapy with low toxicity.

## 2 Experimental section

### 2.1 Materials

Maleimide butanoic acid (MA) was purchased from Aladdin Biochemical Technology Co., Ltd (Shanghai, China). NH2-PEG-NH2 (Mw 3500Da) was obtained from Beijing Kaizheng United Pharmaceutical Technology (Beijing, China). PLGA50/50-COOH (Mw 20000Da) and mPEG5000-PLGA50/50 (Mw 20000Da) were purchased from Daigang Biotechnology Co., Ltd (Jinan, China). The cTMTP1 polypeptide was synthesized by Wuhan Baiyixin Biotechnology Co., Ltd (Wuhan, China). Indocyanine green (ICG, I2633) and monophosphoryl lipid A (MPLA, 699800P) were obtained from Sigma-Aldrich (St. Louis, USA). Coumarin 6 was purchased from MedChem Express (New Jersey, USA). DMEM, McCoy's 5A, 1640 medium, and fetal bovine serum were all purchased from Gbico (California, USA). XPNPEP2 and GAPDH antibodies were purchased from Wuhan Aibo Tek Biotechnology Co., Ltd (Wuhan, China). The cell counting kit-8 (CCK-8) was obtained from Dojindo Chemical Research Institute (Kyushu Island, Japan). The Calcein-AM/PI staining kit was purchased from Shanghai Beyotime Biotechnology Co., Ltd. (Shanghai, China). Cytokines GM-CSF and IL-4 were purchased from Biolegend (California, USA). The CBA multi-factor detection kit was purchased from BD Biosciences (New Jersey, USA). CRT and HSP70 antibodies were obtained from Novus Biologicals (Colorado, USA). Alexa Fluor 594-conjugated secondary antibody was purchased from Invitrogen (California, USA). The immune checkpoint inhibitor anti-PD-L1 antibodies were purchased from BioXcell (New Hampshire, USA). Antibodies against Ki-67, CD4, CD8, Foxp3, and Granzyme B were purchased from Abcam (Cambridge, UK) for Immunohistochemical staining. Antibodies against CD11c, CD80, CD86, CD3, CD4, CD8, CD25, CD44, CD62L, TNF-*α*, IFN-*γ*, Granzyme B, and PD-L1 were all purchased from Biolegend (San Diego, USA) for Flow cytometry analysis. Antibody against PD-L1 was purchased from Abcam (Cambridge, UK), and *β*-actin from ABclonal (Wuhan, China) for western blot. Lyso-Tracker Red was purchased from Servicebio (Wuhan, China). FastPure Cell/Tissue Total RNA Isolation Kit V2, HiScript IV RT SuperMix for qRCR (+gDNA wiper), and ChamQ Universal SYBR qPCR Master Mix were purchased from Vazyme (Nanjing, China).

### 2.2 Preparation and characterization of the TP1-IM micelles

The nanoprodrug MAL-PEG-PLGA were synthesized according to the methods our team previously reported [40]. And the nanomicelles were further synthesized via a one-step nanoprecipitation method. 1 mg cyclicTMTP1 (cTMTP1) peptide and 20 mg of MAL-PEG-PLGA were dissolved in 1 mL N, N-dimethylformamide (DMF) by ultrasonication and reacted at room temperature for 8 h with magnetic stirring, and the cTMTP1-PEG-PLGA were obtained. Then, adding 200 μg free ICG, 150 μg MPLA and different amounts of PBAE (PBAE/ICG mass ratios of 0, 1:1, and 2:1) into the above cTMTP1-PEG-PLGA solution and mixed ultrasonically. The mixture was slowly dripped into 15 mL of deionized water with continuous magnetic stirring for 30 min, and was further centrifuged at 12000 rpm for 30 min at 4 °C. The supernatant was retained for detection of ICG and MPLA encapsulation efficiency by UV–vis Spectrophotometer, and the green precipitate at the bottom of the centrifuge tube was washed twice with deionized water. The precipitate was ultrasonically dissolved with 1 mL of deionized water and stored at 4 °C away from light. The preparation process of TP1-ICG and TP1-MP micelles follows the above method by adding free ICG or MPLA individually. Con-IM micelles were synthesized in a similar manner, except that cTMTP1-PEG-PLGA was replaced by mPEG-PLGA-COOH in the synthesis process.

ICG encapsulation efficiency was measured by UV–vis Spectrophotometer. The absorbance value of the supernatant collected after the first high-speed centrifugation was measured at a wavelength of 784 nm and the ICG content in the supernatant was calculated according to the ICG standard curve. Encapsulation efficiency of ICG in micelles (%) = (Total Amount of ICG - Amount of ICG in Supernatant)/Total Amount of ICG × 100 %. The hydrated particle size representing intensity distribution and zeta potential of micelles was detected by Dynamic Light Scattering (DLS, Zeta Plus, Brookhaven Instruments, USA), and Transmission electron microscopy (JEM-1230, Japan) was employed to characterize the morphology and size of micelles. The UV–visible absorption spectrum of micelles was measured by UV–vis spectrophotometer. MPLA encapsulation efficiency was obtained by UV–vis spectrophotometer in the same way as previously reported [43]. The absorbance value of the supernatant collected after the first high-speed centrifugation was measured at a wavelength of 208 nm, and the MPLA content in the supernatant was calculated according to the MPLA standard curve. The TP1-IM micelles were resuspended in phosphate buffer solution (PBS) or DMEM containing 10 % fetal bovine serum (FBS) individually and stored at 4 °C preventing light. The particle size and dispersibility of TP1-IM micelles were measured by DLS every week for 4 consecutive weeks to determine the stability of nanomicelles. Free ICG, Con-IM, TP1-ICG and TP1-IM nanoparticle aqueous solutions with the same ICG concentration (10 μg/mL) were placed in a cuvette and irradiated with 808 nm NIR laser (2 W/cm^2^) for 5 min. During the irradiation process, the temperature of the above solution was recorded in real time by a NIR thermal imaging camera. PBS was irradiated with NIR laser under the same conditions as a negative control. To evaluate the photothermal conversion efficiency (PCE) of TP1-IM micelles, the temperature changes of TP1-IM solution (10 μg/mL, 1 mL) were recorded every 30 s under NIR treatment with 808 nm laser (2 W/cm^2^). The photothermal conversion efficiency (PCE) was calculated using the method described in the literature [44]. The critical micelle concentration (CMC) of TP1-IM was determined by the HITACHIF-4500 fluorescence spectrophotometer using pyrene as the probe. The pyrene concentration in the nanomicelles solution was 6 × 10^−7^ M. Afterwards, the samples were equilibrated at room temperature overnight. The intensity ratio of I_339_/I_335_ was then recorded to determine the CMC as reported in the previous study [45].

### 2.3 Hemolysis assay

To evaluate the blood compatibility of the TP1-IM *in vitro*, a hemolytic assay was conducted. Fresh blood cells (BCs) were obtained from C57/BL6 mice, and BCs were then centrifuged at 2000 rpm for 5 min. The TP1-IM solution at different concentrations (200, 100, 50, 25, 12.5, and 6.25 μg/mL) was incubated with BCs at the final concentration of 4 % at 37 °C for 1 h. The H_2_O served as the positive control, and PBS as the negative control. After incubation, the supernatants were collected by centrifuging the samples at 2000 rpm for 5 min. The optical density (OD) value of the supernatants was then measured at 540 nm using microplate readers (SpectraMax Paradigm, Molecular Devices, USA). The formula for calculating the hemolysis ratio of the TP1-IM is: hemolysis ratio = (OD value of TP1-IM solution–OD value of PBS)/(OD value of H_2_O–OD value of PBS) × 100 %.

### 2.4 Cell culture and animals

Murine 3T3 and ID8 cell lines were originally obtained from the American type culture collection (ATCC) and were cultured in DMEM medium. Human SKOV3 cells were acquired from the ATCC and were cultured in McCoy's 5A medium. Human A2780 cells were purchased from the European Collection of Authenticated Cell Cultures and were cultured in RPMI-1640 medium, and human CAOV3 cells were obtained from the Characterized Cell line core of MD Anderson Cancer Center (MDACC) and were cultured in DMEM medium. All the medium were supplemented with 10 % FBS, 100 U/mL penicillin and 100 μg/mL streptomycin. All the cells were cultured at 37 °C in a humidified atmosphere incubator containing 5 % CO_2_. Female C57BL/6 mice aged 4–6 weeks were purchased from Beijing Charles River Laboratories and were housed in an SPF-level barrier environment. All the animal experiments were approved by the Experimental Animal Welfare Ethics Committee of Tongji Hospital, Affiliated with Huazhong University of Science and Technology.

### 2.5 The uptake and intracellular localization of micelles *in vitro*

Coumarin 6 (Cou6) was used as a fluorescent probe to replace ICG in Con-IM and TP1-IM nanomicelles to detect the uptake of nanomicelles in cells *in vitro*. Con-CM (control nanoparticles encapsulating Coumarin 6 and MPLA) and TP1-CM (nanoparticles encapsulating Coumarin 6 and MPLA with surface modification of cTMTP1 peptide) micelles containing the same concentration of Coumarin 6 (2.5 ng/mL) were added to ID8 or 3T3 cells and incubated for 30 min, 1 h, 2 h, and 4 h. The fluorescence intensity of Coumarin 6 in the cells was detected by flow cytometer and fluorescence microscope. ID8 or 3T3 cells were cultured in confocal dishes and exposed to TP1-CM in the medium. After incubation for 1 h, 6 h, 10 h, cells were washed with PBS and stained with Lyso-Tracker Red. The degree of lysosomal co-localization was analyzed using CLSM (CLSM610, Ningbo Sunny instruments, China).

### 2.6 Cytotoxicity of TP1-IM *in vitro*

The cytotoxicity of TP1-IM nanomicelles to 3T3 cells and ID8 cells was detected by the CCK-8 assay kit. In brief, the cells were seeded at a density of 6000 cells per well into a 96-well plate and cultured for 24 h. Then, TP1-IM nanomicelles with different ICG concentrations (0, 6.25, 12.5, 25, 50, 100 μg/mL) were individually added to the well plate and incubated for another 24 h. The cells were washed three times with PBS and added with 100 μL serum-free DMEM mixture containing 10 μL CCK-8. After incubation for 3 h in the dark, the optical density (OD) of cells in each treatment group at a wavelength of 450 nm was measured using a microplate reader.

### 2.7 The anti-tumor performance *in vitro*

ID8 cells were seeded into a 96-well plate at a density of 6000 cells/well and cultured for 24 h. The cells were incubated with PBS, Con-IM, TP1-ICG, and TP1-IM micelles containing the same ICG concentration (25 μg/mL) for 2 h. Then, the cells were washed with the fresh medium, irradiated by the 808 nm laser (2 W/cm^2^, 5 min), and further cultured for 24 h. The cell viability was detected by a CCK-8 assay kit or Live/Dead staining kit that live and dead cells could be observed on an inverted fluorescence microscope.

### 2.8 The immune stimulation effect *in vitro*

Bone Marrow-Derived Dendritic Cells (BMDCs) were induced from the tibias and femurs of C57/BL6 mice according to an established method [46]. For the *in vitro* DCs stimulation experiment, the obtained BMDCs were seeded into 12-well plates at a cell density of 2.5 × 10^5^ cells/well and cocultured with PBS, TP1-ICG, free MPLA and TP1-IM for 12 h (ICG = 1 μg/mL, MPLA = 1 μg/mL). Then, the DCs stained with CD11c-FITC, CD80-APC, and CD86-PE antibodies were further analyzed by flow cytometry. For the Transwell Co-culture System assay, BMDCs were cocultured with the residues of ID8 cells after phototherapy treatment with Con-IM, TP1-ICG, and TP1-IM for 12 h. Then, the DCs were collected, stained and analyzed by flow cytometry. Cytometric Bead Assay (CBA) was used to detect the cytokine content in the supernatant of DCs according to the instructions of the kit.

### 2.9 The PD-L1 expression detection induced by PTT *in vitro*

SKOV3, A2780, CAOV3, and ID8 cells were seeded into a 6-well plate at a density of 50,000 cells/well and cultured for 24 h. After incubation with TP1-IM micelles for 2 h, the cells were treated with NIR irradiation (0.5, 1, 2 W/cm^2^, 5 min). After 48 h, the cells with treatment were collected and the PD-L1 expression of treated cells were further detected by western blot, quantitative real-time PCR (qRT-PCR), and flow cytometry. For western blot, cells were lysed to extract the proteins with RIPA buffer, mixed with the protease inhibitor cocktail and phenylmethyl sulfonyl fluoride. Equal amounts of protein samples (20 μg) were separated by 10 % SDS-PAGE and then transferred to polyvinylidene difluoride (PVDF) membranes. After blocking with 5 % bovine serum albumin (BSA) for 1 h, the membranes were incubated with primary antibody at 4 °C overnight. And then after incubated with secondary antibody for 1 h, the samples were detected using an enhanced chemiluminescence (ECL) kit (Advansta, USA) and visualized with a multimode chemiluminescence system (Bio-Rad, USA). For qRT-PCR, total RNA (1 μg) from cells was extracted and cDNA was obtained using FastPure Cell/Tissue Total RNA Isolation Kit V2 and HiScript IV RT SuperMix for qRCR (+gDNA wiper) according to the manufacturer's instructions. Real-time qPCR was performed using ChamQ Universal SYBR qPCR Master Mix according to the manufacturer's instructions for the PCR amplifier. Primer sets were listed in Table S4. For flow cytometry, cells were washed, counted and then incubated with APC-anti-mouse PD-L1 for 30 min. After staining, cells were washed and analyzed on the flow cytometer (Beckman Coulter, USA).

### 2.10 Immunofluorescence

The ICD induced by photothermal therapy was detected by immunofluorescence technique. In brief, ID8 cells seeded into a 24-well culture plate (1 × 10^5^ cells/well) were incubated at 37 °C overnight to reach adherence. ID8 cells were incubated with PBS, Con-IM, TP1-ICG, and TP1-IM micelles (ICG = 25 μg/mL) for 2 h and further treated with 808 nm irradiation (2 W/cm^2^, 5 min). After 12 h, the slices of ID8 cells were fixed with 4 % paraformaldehyde and blocked with 5 % BSA, and then incubated with the primary antibody of anti-HSP70 and anti-CRT (1: 200) overnight at 4 °C. Next, these cells were treated with Alexa Fluor 594-conjugated secondary antibody (1:200) at 37 °C for 1 h. After staining with DAPI for 10 min, the cells were imaged under the confocal laser scanning microscope (Olympus, Tokyo, Japan).

### 2.11 The distribution of TP1-IM *in vivo*

ID8 cells (5 × 10^6^) suspended in PBS were subcutaneously injected into the left flank of each female C57BL/6 mice aged 4–6 weeks. When tumor volume reached about 100 mm^3^, these mice were randomly divided into three groups and injected with Con-IM and TP1-IM through the tail vein, respectively. NIR fluorescence imaging was performed at 1 h, 2 h, 12 h, 24 h and 48 h after administration by IVIS Spectrum Imaging System. For the blocking group, 100 μL of 100 μM cTMTP1 peptide was injected intravenously as a competitive blocking reagent 30 min before. At 48 h, 3 mice from each group were randomly sacrificed, and the main organs (heart, liver, spleen, lungs, kidneys) were isolated and imaged. The exact fluorescent radiant counts of the tissues were measured with the region of interest (ROI) tool in the software. The ROI tool was used to quantify the NIR fluorescence signal intensity of tumor tissue (T) and normal ear tissue (N) in living mice and calculate the ratio between them (T/N value).

### 2.12 Anti-tumor efficacy *in vivo*

Firstly, ID8-luc cells expressed luciferase were established by the lentivirus vector so that the tumor growth could be dynamically detected by the IVIS system through bioluminescence imaging. ID8-luc unilateral subcutaneous tumor-bearing mice were established as described above. When the tumors reached about 100 mm^3^, the mice were randomly divided into 5 groups (n = 9), receiving a single intravenous injection of PBS, Con-IM, TP1-ICG, TP1-IM respectively. After 24 h, the tumor sites were irradiated with an 808 nm laser (2 W/cm^2^) for 5 min. The tumor size and surviving number of mice were monitored every 2 days for 90 days. The mice were killed when the tumor volume reached 1000 mm^3^. The tumor volume (V) was calculated using the formula: V = 1/2 ab^2^, where a and b are the long and short axes of the tumor, respectively.

For the tumor metastasis mice models, ID8-luc cells (5 × 10^6^) were firstly subcutaneously injected into the left hip of each female C57BL/6 mice aged 4–6 weeks, and when the tumor volume reached 100 mm^3^, ID8-luc cells were inoculated into the right hip or abdominal cavity of the mice to simulate tumor metastasis. On the day of ID8-luc cell secondary inoculation, mice in different groups were injected intravenously with PBS, Con-IM, TP1-ICG, TP1-IM, and TP1-IM, respectively. On day 0, the primary tumors were irradiated under 808 nm (2 W/cm^2^, 5 min) or surgically removed. And on days 1, 4, and 7 after NIR irradiation, mice in the anti-PD-L1 treatment groups were intraperitoneally injected with 100 μL of anti-PD-L1 antibody. The tumor size was measured with a vernier calliper for 10 weeks or detected by bioluminescence imaging for 8 weeks.

### 2.13 Immune response analysis for tumor metastasis

For the analysis of systemic immune activation, three mice in each group were sacrificed, and spleens and tumor-draining lymph nodes (TDLNs) were isolated and prepared into single-cell suspensions. The cells were stained with anti-CD3-FITC, anti-CD4-APC, anti-CD8-Percp/Cyanine5.5, anti-CD25-PE, IFN-*γ*-BV421, Granzyme B-BV605, TNF-*α*-BV650, and further analyzed by flow cytometry.

The infiltrating T lymphocytes in secondary tumor tissues were detected by immunohistochemistry. Briefly, the tumor tissue slides were dewaxed, hydrated, antigen retrieved, inactivated, and blocked according to standard procedures. After that, the tissue slides were incubated overnight at 4 °C with the primary antibody for anti-CD3, anti-CD4, anti-CD8, anti-Foxp3 and anti-Granzyme B, followed by being incubated with HRP-labeled secondary antibody. After being stained with DAB, the typical images were imaged with an Olympus BX53 microscope (Olympus, Tokyo, Japan).

### 2.14 Memory T cells

To investigate the immune memory T cells, three mice from each treatment group were killed on day 40, and spleen and orbital blood were isolated and collected. The spleens were isolated and prepared into single-cell suspensions. The cells were stained with anti-CD3-FITC, anti-CD4-APC, anti-CD8- Percp/Cyanine5.5, anti-CD44-PE, anti-CD62L-Percp/Cyanine5.5 or anti-CD62L-APC antibodies and further analyzed by flow cytometry. Cytometric Bead Assay (CBA) was used to detect the cytokine content in the supernatant of blood according to the instructions of the kit.

### 2.15 Statistical analysis

Data were presented as the mean ± standard deviation (SD). Differences among groups were analyzed by one-way ANOVA or unpaired two-sided Student's t-test using GraphPad Prism 8.0 software. Differences in survival time were analyzed using the log-rank test in Kaplan-Meier survival analysis. The *P*-value of <0.05 was considered statistically significant.

## 3 Results and discussion

### 3.1 Preparation and characterization of TP1-IM

**Fig. 1:**
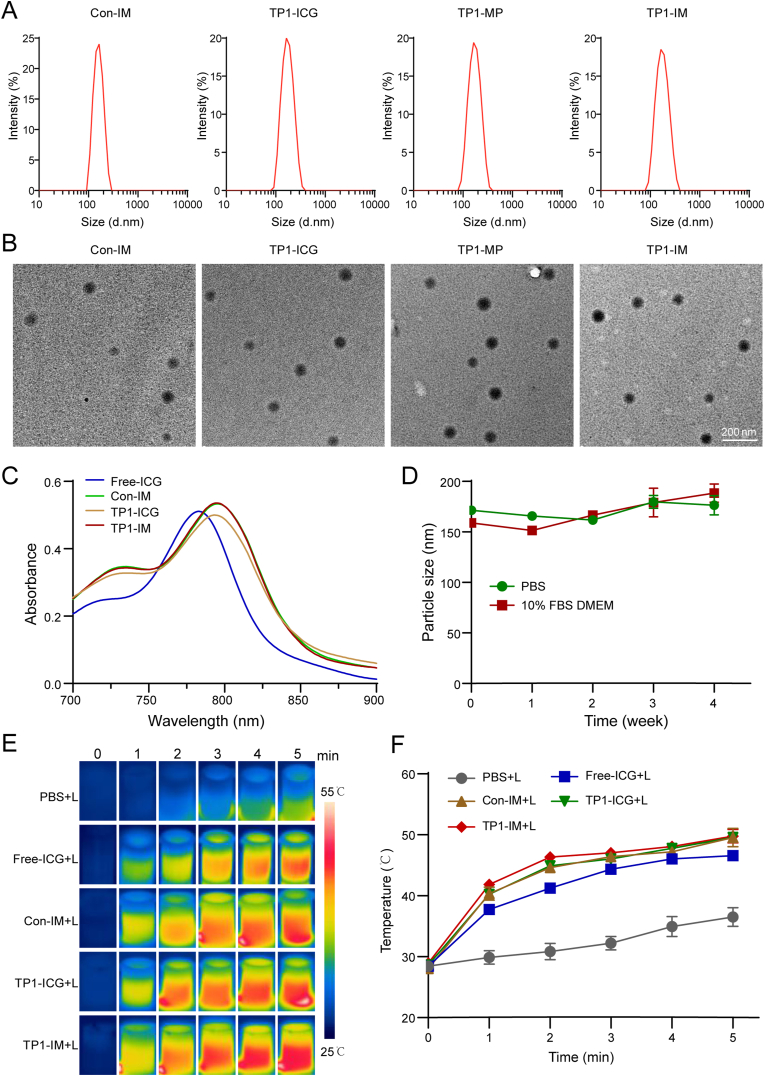
Characterization of Con-IM, TP1-ICG, TP1-MP and TP1-IM micelles. (A) Diameter distribution of the four micelles were measured by DLS. (B) TEM images of four micelles. Scale bar: 200 nm. (C) UV–vis absorption spectra of free ICG and the micelles were measured with the same ICG concentration (3 μg/mL). (D) The stability of TP1-IM in PBS and DMEM containing 10 % FBS was determined within 4 weeks at 4 °C. (E) Infrared thermal imaging representation of free ICG and micelles with the same ICG concentration (10 μg/mL) under NIR irradiation (808 nm, 2 W/cm^2^) within 5 min. (F) The temperature changes of free ICG and micelles within 5 min under NIR irradiation (808 nm, 2 W/cm^2^).

TP1-IM micelles were synthesized using the one-step nanoprecipitation method. cTMTP1-PEG-PLGA was first prepared by linking cyclic TMTP1 polypeptide (cTMTP1) and MAL-PEG-PLGA via thiol-maleimide click chemistry, as reported previously [40]. Then, ICG and MPLA were encapsulated into the core of the PEG-PLGA using the nanoprecipitation method. However, as the results shown in Table S1, the encapsulation efficiency of ICG in TP1-IM micelles was too low to generate enough energy to kill tumor cells. The PBAE-g-β-CD copolymer (PBAE) was synthesized via Michael addition polymerization as our previous work described [47]. The structure of PBAE was confirmed by proton nuclear magnetic resonance (Fig. S1). As a cationic material, PBAE could adsorb ICG by attracting positive and negative charges, thereby increasing the ICG content in micelles. As the content of PBAE increased, the encapsulation efficiency of ICG in TP1-IM micelles was also enhanced. When the mass ratio of PBAE to ICG was 2:1, the encapsulation efficiency of ICG in TP1-IM micelles could be enhanced to 78.57 ± 5.41 %, but no significant particle size change was observed (Table S1). As reported in previous studies [48,49], the possible driving force for this enhanced encapsulation efficiency lies primarily in the electrostatic interactions between the positively charged PBAE and the negatively charged ICG molecules. The cationic nature of PBAE allows it to effectively interact with the anionic ICG through electrostatic forces, facilitating the encapsulation of ICG into the hydrophobic core of the micelles. This electrostatic attraction helps stabilize the ICG within the micellar structure, thus improving its loading capacity. Moreover, PBAE could also contribute to the formation of more stable nanoparticles, thereby reducing the leakage of ICG during the storage or administration process. Furthermore, the encapsulation efficiency of MPLA in TP1-IM was increased from 61.29 ± 0.30 % to 90.33 ± 0.37 % with the addition of PBAE (Table S2). The potential mechanism behind this phenomenon may lie in electrostatic interactions for the negative charges of the MPLA [50,51]. Following the preparation method, Con-IM (control nanoparticles without cTMTP1 modification), TP1-ICG (nanoparticles modified with cTMTP1 but only loaded with ICG) and TP1-MP (nanoparticles modified with cTMTP1 but only loaded with MPLA) were successfully prepared. Nanoparticles containing ICG presented green appearance as shown in Fig. S2. The DLS measurements of Con-IM, TP1-ICG, TP1-MP, and TP1-IM micelles showed single-distribution peaks, with corresponding average hydrated particle sizes of 157.30 ± 4.80 nm, 167.20 ± 8.20 nm, 167.50 ± 8.70 nm and 165.50 ± 8.20 nm (Fig. 1A and Table S3). The corresponding surface zeta potential of these four micelles was −18.99 ± 2.47 mV, −13.25 ± 1.34 mV, −23.97 ± 2.41 mV and −9.93 ± 1.52 mV, respectively (Table S3). Due to the introduction of PBAE and modification of TMTP1, the negative charge of TP1-ICG and TP1-IM nanoparticles was weakened but still negative. All four micelles had a uniform spherical shape, as observed by Transmission Electron Microscopy (TEM). The size of the four micelles under TEM was approximately 80–100 nm, slightly smaller than the hydrated particle size (Fig. 1B). Ultraviolet–visible absorption spectra of the nanoparticles were further measured and we found that the peak absorbance of Con-IM, TP1-ICG and TP1-IM was red-shifted from 780 nm to 810 nm compared with free ICG (Fig. 1C). The slight increase in absorption at the 810 nm wavelength might be due to the hydrophobic environment in which the ICG molecules resided [52,53]. The results also indicated that ICG encapsulated into nanoparticles retained its good near-infrared fluorescence properties. The TP1-IM showed remarkable stability without significant size changes in PBS and 10 % FBS DMEM for 4 weeks at 4 °C, which ensured their feasibility for further investigation (Fig. 1D). To further evaluate the photothermal ability of ICG-containing nanoparticles, an infrared thermal imaging camera was used to record the temperature changes within 5 min under NIR irradiation (808 nm, 2 W/cm^2^) of the micelles with the same ICG concentration (10 μg/mL). The results indicated that the photothermal ability of ICG-loaded micelles was significantly better than that of PBS, presented by the temperature rising from 28.5 °C to 50 °C (Fig. 1E–F). And as shown in Fig. S3A, the photothermal conversion efficiency (PCE) of TP1-IM micelles under 808 nm laser irradiation was determined to be 55.73 %. After five cycles of laser on/off exposure, the solution temperature of TP1-IM remained stable, suggesting that the material possesses strong optical and thermal durability (Fig. S3B). The hemolysis test result showed that compared to the positive control of H_2_O, TP1-IM micelles did not display a visible hemolytic effect at different concentrations (Fig. S3C). And the CMC of TP1-IM was determined as 11.52 μg/mL (Fig. S3D). These results demonstrated that ICG-loaded micelles possess good photothermal properties and can be used in subsequent photothermal therapy experiments.

### 3.2 Cellular uptake, intracellular localization, cytotoxicity and photothermal killing effect of TP1-IM *in vitro*

**Fig. 2:**
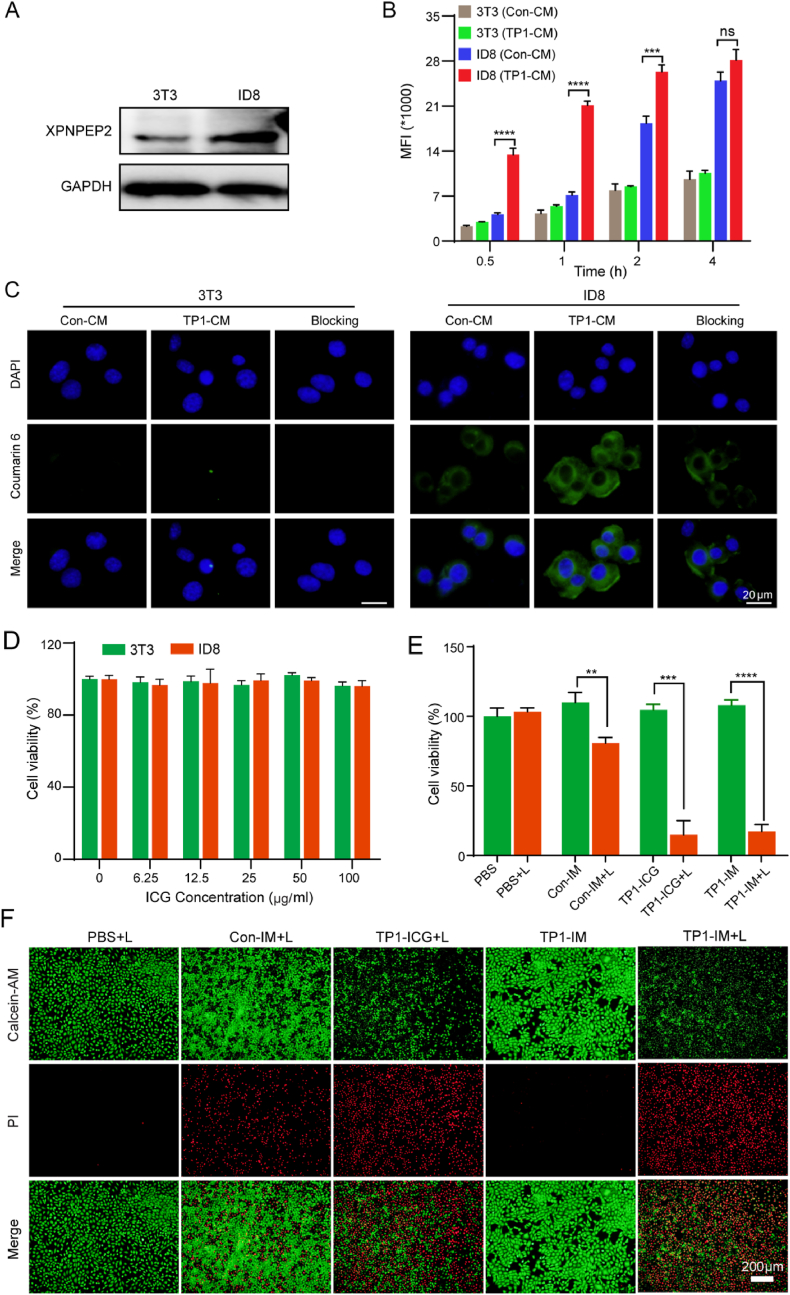
*In vitro* cellular uptake and photothermal killing effect of TP1-IM micelles. (A) The expression level of XPNPEP2 protein in 3T3 and ID8 cells was detected by western blot. (B) Flow cytometry analysis of the cellular uptake of nanoparticles by 3T3 cells and ID8 cells after incubating with Con-CM or TP1-CM (Cou6 2.5 ng/mL) for 30 min, 1 h, 2 h, or 4 h, respectively. (C) The cellular uptake in 3T3 and ID8 cells after incubation with Con-CM and TP1-CM for 2 h was observed under the fluorescence microscope. The blocking group was to add 10 times cTMTP1 polypeptides for 30 min before adding TP1-CM into cells. Scale bar: 20 μm. (D) The cytotoxicity of TP1-IM with different ICG concentrations was detected by the CCK-8 kit when they were incubated with 3T3 and ID8 cells for 24 h, respectively. (E) The cell viability on ID8 cells incubated with Con-IM, TP1-ICG, and TP1-IM for 2 h and treated under NIR irradiation (808 nm, 2 W/cm^2^, 5 min). (F) The photothermal killing effect of micelles on ID8 cells under NIR irradiation (808 nm, 2 W/cm^2^, 5 min) was detected by Calcein-AM/PI staining kit. Green fluorescence: Calcein-AM dye, living cells; Red fluorescence: PI dye, dead cells. Scale bar: 200 μm. n.s: not significant, ∗∗: *p* < 0.01, ∗∗∗: *p* < 0.001, ∗∗∗∗: *p* < 0.0001. (For interpretation of the references to colour in this figure legend, the reader is referred to the Web version of this article.)

The tumor specificity of the TP1-IM micelles *in vitro* was further investigated in mouse-derived ovarian cancer cells ID8 and normal mouse embryonic fibroblasts cells 3T3. As one of the potential receptors of TMTP1 polypeptide, the XPNPEP2 expression on the two cell lines was determined by western blot. As shown in Fig. 2A, the expression level of XPNPEP2 in ID8 cells was obviously increased than that of 3T3 cells. And Coumarin 6 was used to trace nanomicelles *in vitro*. When the two cells were incubated with Con-CM micelles and TP1-CM micelles for 4 h, faster and higher accumulation was recorded in ID8 cells than in 3T3 cells, and TP1-CM micelles showed significant tumor cell preference than Con-CM micelles in ID8 cells (Fig. 2B). After co-incubation for 4 h, the uptake of Con-CM and TP1-CM were not obviously different in ID8 cells, and then 2 h was chosen as the incubation time point for subsequent experiments *in vitro* (Fig. 2B). Further fluorescence microscope imaging revealed that nanomicelles were accumulated in the cytosol of 3T3 cells and ID8 cells after incubation for 2 h. In contrast to Con-CM, the uptake of TP1-CM was significantly increased in ID8 cells (Fig. 2C). To further evaluate whether the uptake of TP1-CM in ID8 cells was mediated by the cTMTP1 peptide, ID8 cells were pretreated with cTMTP1 peptide for 30 min before incubation with TP1-CM. As shown in Fig. 2C, the uptake of TP1-CM micelles in ID8 cells was mostly blocked by cTMTP1 peptide. Furthermore, the CLSM was used to examine the intracellular trafficking profile of the TP1-CM micelles, and the result indicated that the TP1-CM micelles entered the cells after 1 h of incubation. Six hours later, most of the TP1-CM were visualized at the lysosome, and the nanomicelles started to escape from the lysosome after 10 h of incubation (Fig. S3E). Given its excellent OC-targeted drug delivery ability, these results showed that TP1-CM micelles could preferentially deliver their drugs into the cytosol of ID8 cells, probably through an XPNPEP2 dependent pathway.

Biocompatibility was important for the clinical transformation of drugs. Cell viability was further evaluated by the CCK-8 assay in ID8 cells and 3T3 cells. Both cells were incubated with different concentrations of TP1-IM for 24 h. As the result showed in Fig. 2D, the cell viability remained above 95 % when the ICG concentration of TP1-IM micelles reached 100 μg/mL. The results indicated that the TP1-IM micelles had good biocompatibility and were virtually non-cytotoxic. When exposed to the NIR irradiation (808 nm, 2 W/cm^2^, 5 min), TP1-ICG and TP1-IM displayed significantly stronger tumor cell killing effects than Con-IM, and the cell viability decreased to 14.9 % and 17.3 %, respectively (Fig. 2E). A calcein-AM/PI staining experiment was further conducted to observe the apoptosis of cells visually. Calcein-AM stains live cells in green, while propidium iodide (PI) stains dead cells in red. Cell death was obviously observed in TP1-ICG + L and TP1-IM + L groups (Fig. 2F). These results demonstrated that the TP1-IM micelles are excellent photothermal agents under 808 nm laser irradiation.

### 3.3 Immune stimulation effect induced by TP1-IM *in vitro*

DCs, as the main antigen-presenting cells, play a crucial role in initiating and regulating innate and adaptive immunity. Immature DCs collect antigens from surrounding body fluids and then transform into mature DCs during the process of migration from peripheral tissues to nearby draining lymph nodes, and process antigens into peptides. Mature DCs present peptide-major histocompatibility complexes to T cell surface receptors, which in turn activate T cells. The co-stimulatory molecules CD80 and CD86 upregulation are classic markers of the DCs maturity. MPLA, as a chemical derivative of lipopolysaccharides, could induce strong immune-stimulating activity and was often used as an adjuvant for vaccines. Thus, the immune stimulation effect of TP1-IM micelles was first examined *in vitro*. BMDCs were cocultured with PBS, TP1-ICG, free MPLA and TP1-IM micelles for 12 h, respectively, and DCs maturation was examined by flow cytometry. As shown in Fig. S4A–4B, the percentage of mature DCs (CD11c^+^CD80^+^CD86^+^) induced by TP1-IM was considerable to that of free MPLA with the same dose, while TP1-ICG without MPLA had no obvious immune stimulation effect to DCs. Meanwhile, the DCs-secreted immune-related cytokines such as interleukin 12 (IL-12p70) and tumor necrosis factor α (TNF-α), which were also indicators of DCs activation, were measured by flow cytometry. It was found that the secretion levels of IL-12p70 and TNF-α from DCs were obviously enhanced after TP1-IM treatment to levels similar to those post-treatment with free MPLA (Fig. S4C–4D). Thus, TP1-IM micelles themselves could act as a strong immune adjuvant.

**Fig. 3:**
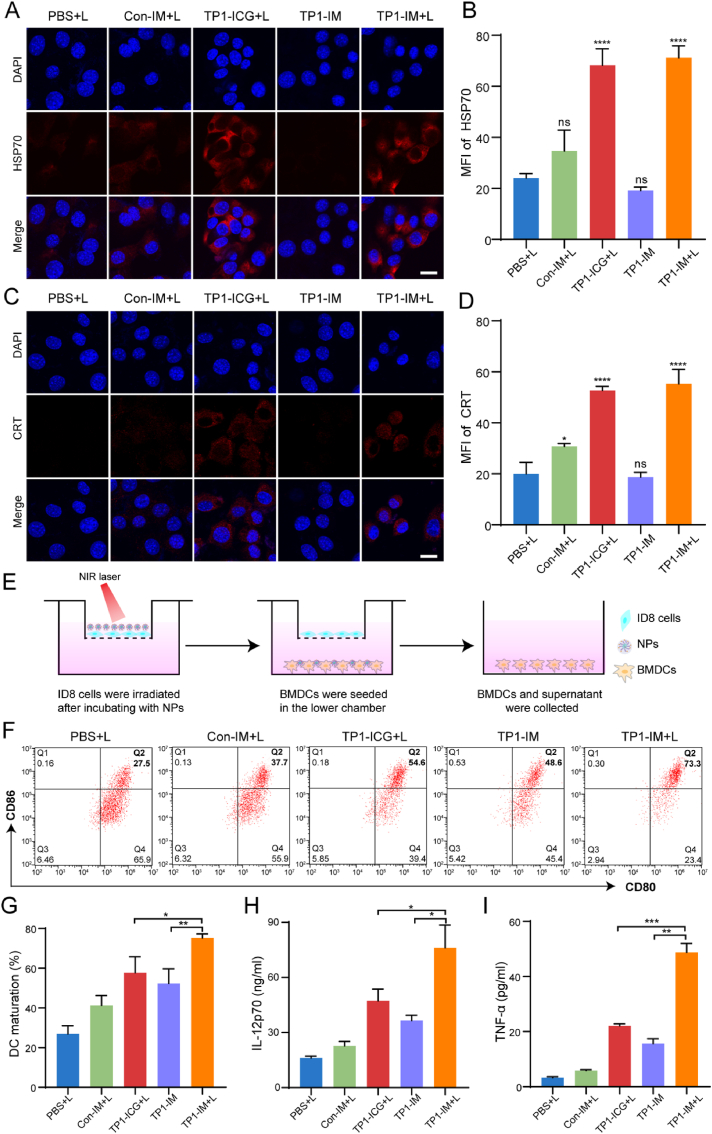
ICD induction and DCs maturation induced by TP1-IM under NIR irradiation *in vitro*. (A–B) Confocal images of ID8 cells with immunofluorescence staining of HSP70 (A) and the comparison of HSP70 expression levels after different treatments for 12 h (B), ID8 cells were incubated with Con-IM, TP1-ICG and TP1-IM for 2 h and treated under NIR irradiation (808 nm, 2 W/cm^2^, 5 min). Scar bar:15 μm. (C–D) Confocal images of ID8 cells with immunofluorescence staining of CRT (C) and the comparison of CRT expression levels after different treatments for 12 h (D). Scar bar:15 μm. (E) Schematic illustration of Transwell co-culture system experiment. **(**F) Flow cytometry analysis of the proportion of DCs maturation (CD11c^+^CD80^+^CD86^+^) in Transwell co-culture system. **(**G) Quantitative analysis of DCs maturation in Transwell co-culture system. (H–I) The levels of IL-12p70 and TNF-α in the supernatant of DCs after different treatments for 12 h. n.s: not significant, ∗: *p* < 0.05; ∗∗: *p* < 0.01; ∗∗∗: *p* < 0.001; ∗∗∗∗: *p* < 0.0001.

It was reported that PTT could produce an antitumor immune response by inducing ICD. After PTT treatment, necrotic or apoptosis cells could release TAAs and express Damage-Associated Molecular Patterns (DAMPs). DAMPs bind to the corresponding receptors or ligands on DCs to activate the transition of immature DCs to mature phenotypes, and mature DCs further engulf TAAs and present TAAs to T cells. In this process, Heat Shock Protein 70 (HSP70) was ectopic from the nucleus to the cell surface, and Calreticulin (CRT) was ectopic to the cell surface from the endoplasmic reticulum, both of which could exert their immune activation by binding to DCs. Hence, we examined the expression of HSP70 and CRT on ID8 cells with different treatments by immunofluorescence staining technique. Under 808 nm laser irradiation, TP1-ICG and TP1-IM treatments significantly upregulated the expression of HSP70 and CRT on ID8 cells, indicating photothermal ablation of ID8 cells could induce ICD efficiently, thus triggering downstream immune responses (Fig. 3A–D).

A transwell system was employed to assess the immune stimulation effect of TP1-IM under laser irradiation. The experimental design was shown in Fig. 3E. Firstly, ID8 cells were planted in the upper chamber and irradiated with 808 nm laser (2 W/cm^2^) for 5 min after coculturing with PBS, Con-IM, TP1-ICG and TP1-IM containing the same ICG concentration (25 μg/mL). Then, the treated ID8 cells were cocultured with the BMDCs planted in the lower chamber for 12 h. Flow cytometry was used to detect the mature ratio of DCs and secreted cytokine levels after different treatments. Compared with the control group, the TP1-ICG + L group could significantly promote the maturation of BMDCs, and the TP1-IM + L group exhibited the strongest role in promoting DCs maturation (Fig. 3F–G). It was found that the secretion levels of IL-12p70 and TNF-α from cell supernatant were mostly enhanced after TP1-IM with laser irradiation treatment (Fig. 3H–I). The results indicated that with the help of MPLA-mediated immune adjuvant effects, TP1-IM micelles prepared in this study could effectively activate BMDCs under NIR laser irradiation, laying the foundation for the anti-tumor immune effects of the micelles *in vivo*.

### 3.4 The targeted tumor imaging and photothermal effect of TP1-IM *in vivo*

**Fig. 4:**
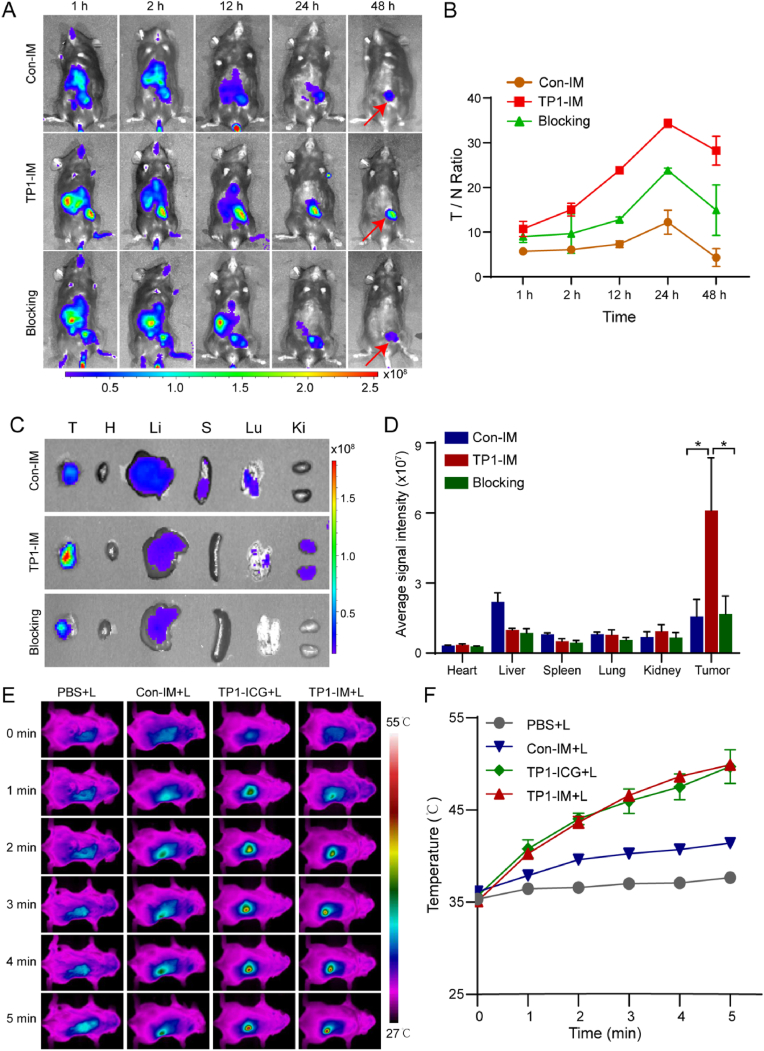
The targeted tumor imaging and photothermal ability of TP1-IM *in vivo*. (A) Representative NIR fluorescence images of ID8 tumor-bearing mice at 1 h, 2 h, 12 h, 24 h, and 48 h after intravenous injection of Con-IM and TP1-IM. For the blocking group, 100 μL of 100 μM cTMTP1 peptide was injected intravenously as a competitive blocking reagent 30 min before. (B) The fluorescence intensity ratio between tumor areas and normal tissues (ear) in each group of mice (T/N ratio). (C) *Ex vivo* fluorescence images of major organs and tumors excised from mice at 48 h post-injection Con-IM and TP1-IM, respectively. (D) Quantitative analysis of MFI of tumor tissues and major organs at 48 h after drug administration. (E) Representative infrared thermal imaging images of tumor areas in tumor-bearing mice under NIR laser irradiation after different treatment. (F) Temperature curves of tumor areas in tumor-bearing mice within 5 min under NIR laser irradiation. ∗: *p* < 0.05.

To investigate the targeting ability of TP1-IM micelles to OC *in vivo*, the ID8 tumor-bearing mice were established by subcutaneously injecting ID8 cells into the left flank of female C57/BL6 mice. When the tumor volume reached 100 mm^3^, Con-IM and TP1-IM were intravenously injected, and NIR fluorescence imaging was recorded at 1, 2, 12, 24 and 48 h after drug administration. It was clearly observed that TP1-IM exhibited gradually clear and specific fluorescent signals at the tumor site from 1 h to 48 h (Fig. 4A). Strong NIR fluorescence signals could still be observed at the tumor site even 48 h after TP1-IM injection, indicating that TP1-IM could not only specifically target ID8 tumors but also be retained in the tumor site for a long time. In the TP1-IM + cTMTP1 treatment group (Blocking group), the intensity of fluorescent signal at the tumor site was significantly weakened than that in the tumor-bearing mice group injected with TP1-IM, indicating that the pre-injected cTMTP1 peptides in tumor-bearing mice could partially block the binding of TP1-IM to ID8 cells and further demonstrating the OC targeting properties of cTMTP1 peptides (Fig. 4A). The fluorescent signal ratio of tumor to normal tissue (T/N) was measured and calculated quantitatively. As shown in Fig. 4B, The T/N ratio of TP1-IM reached its peak 24 h after administration and was significantly higher than that of Con-IM. To further investigate the distribution of micelles *in vivo*, mice in each group were sacrificed 48 h after administration. The tumor and major organ tissues (heart, liver, spleen, lung, kidney) were isolated for further fluorescence NIR imaging *ex vivo*. As shown in Fig. 4C–D, TP1-IM was primarily metabolized through the liver. Compared with Con-IM, a stronger fluorescent signal was observed in the tumor site of tumor-bearing mice treated with TP1-IM, which was 3.88 times higher than that of Con-IM micelles. All these results presented the excellent targeting ability of TP1-IM in ID8 tumor-bearing mice.

In order to evaluate the photothermal ability of TP1-IM *in vivo*, the tumor sites of ID8 tumor-bearing mice were irradiated under 808 nm laser for 5 min at 24 h after drug injection intravenously, and an IR thermal imaging camera was chosen to real-time monitor the temperature of tumor site. As shown in Fig. 4E–F, under NIR laser irradiation, the temperature of the tumor site of mice injected with TP1-ICG and TP1-IM rapidly rose to approximately 44 °C within 2 min and reached approximately 49 °C after 5 min of irradiation, which was significantly higher than that induced by the Con-IM. Meanwhile, the tumor temperature had no obvious change by treating with PBS injection under laser irradiation. The result indicated that the excellent tumor targeting of TP1-IM micelles ensured good photothermal conversion ability in ID8 tumor-bearing mice.

### 3.5 Anti-tumor effect of TP1-IM *in vivo*

**Fig. 5:**
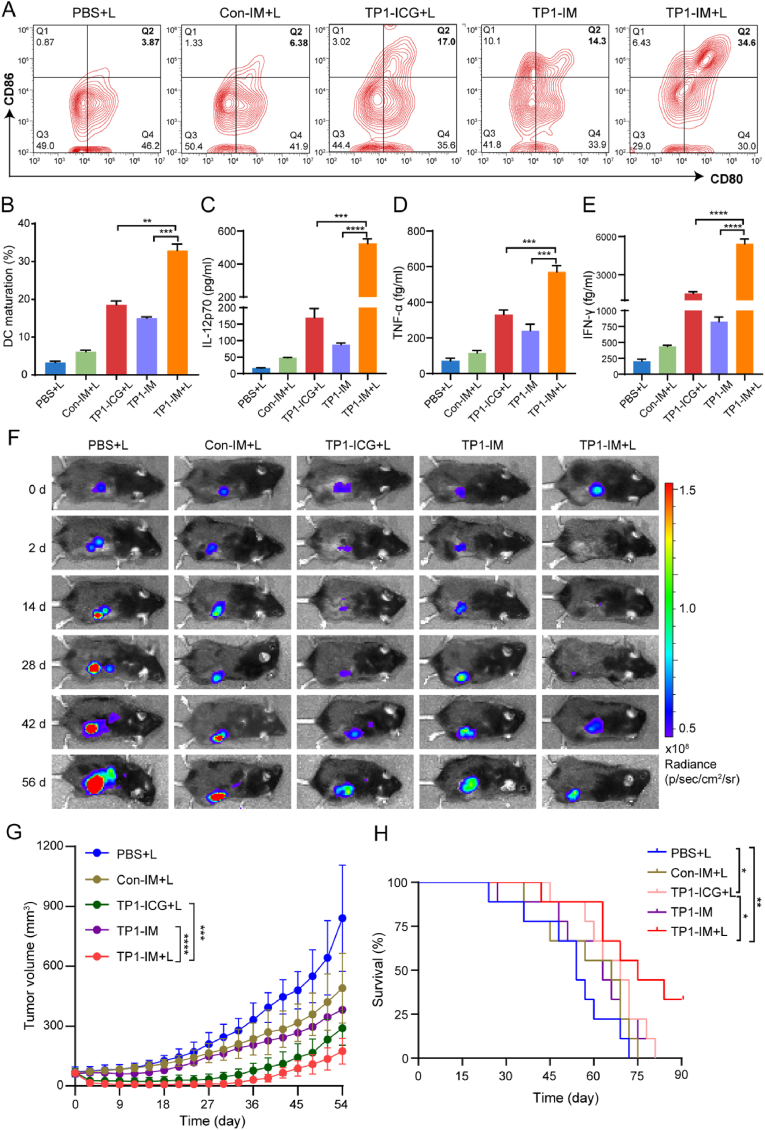
DCs maturation and anti-tumor effect of TP1-IM *in vivo*. (A) Flow cytometry analysis of DCs maturation (CD11c^+^CD80^+^CD86^+^) induced by TP1-IM-based PTT on mice bearing ID8 tumors. The tumor sites were irradiated under NIR irradiation, and TDLNs were isolated and prepared into single cells for analysis after 3 days. (B) Quantitative analysis of DCs maturation in TDLNs on mice bearing ID8 tumors after different treatments for 3 days. (C–E) The expression levels of IL-12p70, TNF-α and IFN-γ in the serum of tumor-bearing mice were detected by CBA. (F) Representative bioluminescence images of ID8 tumor-bearing mice after different treatments. (G) Tumor growth curves of tumor-bearing mice after different treatment. (H) Survival curve of tumor-bearing mice in each group after different treatments. ∗: *p* < 0.05, ∗∗: *p* < 0.01, ∗∗∗: *p* < 0.001, ∗∗∗∗: *p* < 0.0001.

We further evaluated the immunogenicity of PTT with TP1-IM micelles *in vivo* on ID8 subcutaneous tumor-bearing mice. The mice were intravenously injected with PBS, Con-IM, TP1-ICG, and TP1-IM and the tumor site was treated with NIR laser irradiation (808 nm, 2 W/cm^2^) for 5 min at 24 h after drug administration intravenously. Three days after PTT treatment, TDLNsof mice were isolated, and the proportion of mature DCs in TDLNs was detected by flow cytometry. As shown in Fig. 5A–B, compared with the PBS + L group, both the TP1-ICG + L and TP1-IM groups could significantly promote the maturation of DCs, indicating that PTT and adjuvants could both activate DC cells. Notably, TP1-IM treatment-based PTT could induce a much higher proportion of DCs maturation than that of the TP1-ICG + L group without MPLA or the TP1-IM groups in the absence of laser irradiation. Meanwhile, we detected the levels of various cytokines, including IL-12p70, TNF-α, and IFN-γ in the serum of mice with different treatments by flow cytometry. As shown in Fig. 5C–E, the proportion of cytokine IL-12p70, TNF-α, and IFN-γ in the TP1-IM with PTT was significantly enhanced than in other groups. The results indicate that TP1-IM micelles could stimulate immune activation in ID8 tumor-bearing mice after PTT and may be conducive to enhancing the tumor suppression effect.

Next, we evaluated the anti-tumor activity of TP1-IM-based PTT *in vivo*. The subcutaneous ID8 tumor-bearing mice were intravenously injected with PBS, Con-IM, TP1-ICG, and TP1-IM and treated with NIR laser irradiation (808 nm, 2 W/cm^2^) for 5 min at 24 h post-injection. The tumor volume growth was monitored by bioluminescence imaging every two weeks and measured by a vernier caliper every three days simultaneously. The survival status was also recorded to assess the therapeutic efficacy. As shown in Fig. 5F, the fluorescence signal in the tumor site of the TP1-ICG + L and TP1-IM + L groups decreased significantly after 2 days of treatment, indicating that TP1-ICG and TP1-IM-based PTT could rapidly and effectively destroy ID8-tumors. Compared with the PBS + L group, the Con-IM, TP1-ICG + L and TP1-IM groups exhibited certain tumor inhibition effects. In contrast, the TP1-IM + L treatment group presented a much better tumor suppressive effect and could almost completely eliminate tumors on the 6th day after NIR laser irradiation, and the tumor suppressive effect even lasted until the 27th day. Unfortunately, the tumor began to recur after the 27th day of treatment (Fig. 5F–G). In addition, the survival rate of each group was also analyzed. As shown in Fig. 5H, compared with other groups, TP1-IM-based PTT significantly prolonged the survival time of ID8 tumor-bearing mice.

The mice were sacrificed to isolate tumor tissues for hematoxylin-eosin (HE) and TUNEL staining analysis on the 6th day after PTT. As shown in Fig. S5, compared with other groups, HE staining in the TP1-ICG + L and TP1-IM + L groups showed extensive pyknosis and fragmentation of tumor cell nuclei. TUNEL staining showed a significant increase in apoptosis and necrotic cells in the tumor tissues of TP1-ICG + L and TP1-IM + L groups. In addition, the major organs (heart, liver, spleen, lung, kidney) were also separated for HE staining. It was found that the tissues in different groups showed similar tissue structures, indicating that the treatment had no obvious toxicity *in vivo* (Fig. S6). The results indicated that the TP1-IM-based PTT had a rapid and significant killing effect on ID8 tumors and could effectively inhibit tumor growth by synergizing with immune adjuvant MPLA.

### 3.6 *In vivo* assessment of inhibition effect against metastasis by TP1-IM combined with PD-L1 blockade

Metastasis was an important reason for the poor prognosis of ovarian cancer patients, and it was rather difficult to prevent and suppress tumor metastases while treating the primary tumor with traditional treatment such as surgery and chemotherapy. Previous studies have demonstrated the synergistic immunotherapeutic and chemotherapeutic efficacy of nanovesicles in inhibiting the tumor growth [54,55]. The immune checkpoint inhibitor anti-PD-L1 was reported to break tumor immune tolerance by blocking the PD-1/PD-L1 signaling pathway and restoring the killing function of tumor-specific T cells, and then exerting an anti-tumor effect [56]. As shown in Fig. S7A–C, the expression level of PD-L1 on SKOV3, A2780, CAOV3, and ID8 cells was upregulated with irradiation under 808 nm (0.5, 1, and 2 W/cm^2^). These results suggested that PTT could induce PD-L1 expression in ovarian cancer cells, indicating the foundation for the combination therapy of PTT and anti-PD-L1 treatment. Therefore, PD-L1 blockade therapy was introduced in our study, aiming to improve the inhibiting effect on tumor metastasis.

**Fig. 6:**
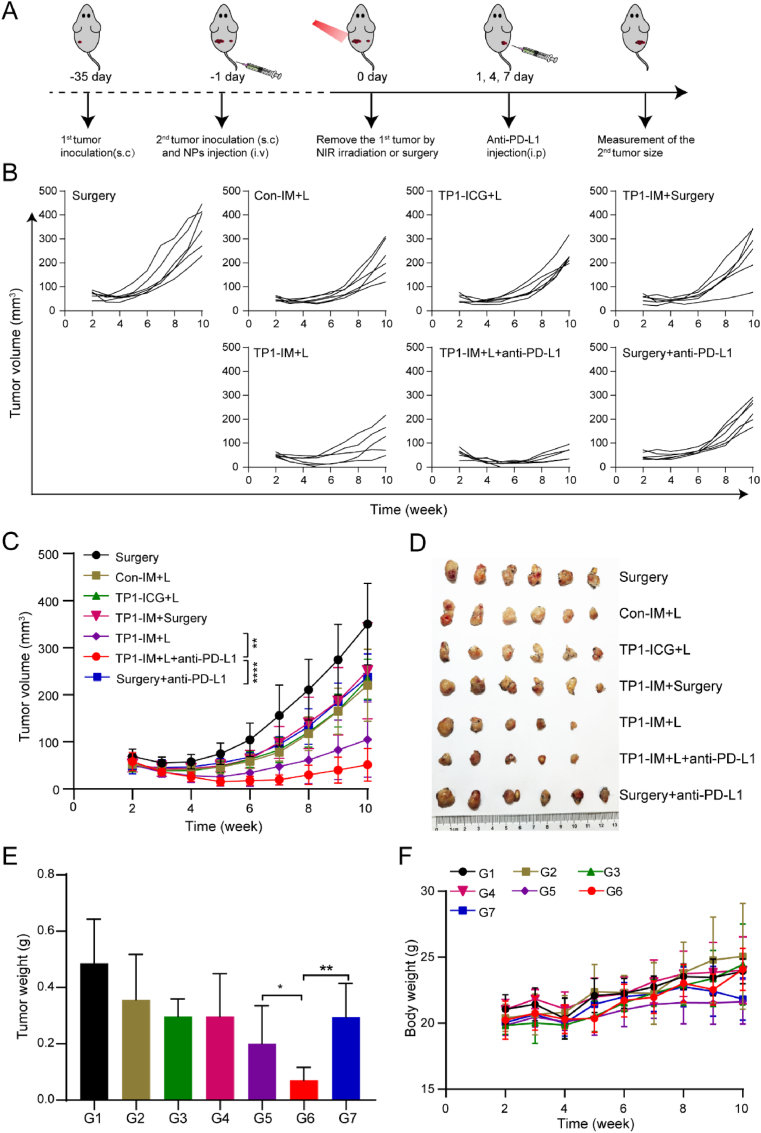
Inhibition of OC metastasis by TP1-IM-based PTT combined with PD-L1 checkpoint blockade. (A) Schematic illustration of the therapeutic schedule of bilateral subcutaneous tumor models. (B) Individual distant tumors progression curves of tumor-bearing mice after different treatments. (C) Growth curves of distant tumors after different treatment. (D) Photographs of distant tumors after different treatment. (E) Tumor weight of each group. (F) Body weight change curves of mice after the treatment. ∗: *p* < 0.05, ∗∗: *p* < 0.01, ∗∗∗∗: *p* < 0.0001.

As shown in Fig. 6A, a bilateral OC mice model was established by subcutaneously injecting ID8 cells into both hips of C57/BL6 mice at 35 days and 1 day separately before treatment. The first and second tumors were applied to simulate the primary and secondary metastasis tumors, respectively. And we randomly divided these mice into seven groups: (G1) Surgery; (G2) Con-IM + L; (G3) TP1-ICG + L; (G4) TP1-IM + Surgery; (G5) TP1-IM + L; (G6) TP1-IM + L + anti-PD-L1; (G7) Surgery + anti-PD-L1; +L represented that the group treated with NIR laser irradiation (808 nm, 2 W/cm^2^, 5 min). After 24 h of drug administration intravenously, the primary tumors of mice in each group were removed through laser irradiation or surgery on day 0. Mice in the G6 and G7 were intraperitoneally injected with the anti-PD-L1 antibodies on days 1, 4, and 7.

Tumor growth of the secondary tumors and the body weight of mice were recorded every week for 10 weeks. As shown in Fig. 6B–C, compared with the Surgery group, the Con-IM + L, TP1-ICG + L, TP1-IM + Surgery, and Surgery + anti-PD-L1 monotherapy groups had certain inhibition effects on distal tumor growth but were not particularly significant. In contrast, the TP1-IM + L treatment group combined PTT and MPLA could obviously inhibit the growth of distal tumors. It was worth noting that the TP1-IM + L + anti-PD-L1 treatment group exhibited the strongest inhibitory effect on distal tumor growth compared to all other groups. At the end of the observation, the tumors of mice in each group were excised and weighed. The TP1-IM + L + anti-PD-L1 group presented the smallest tumor size and the lightest tumor mass (Fig. 6D–E). As presented in Fig. 6F, no significant changes in body weight were observed in mice from all groups during the treatment, indicating that the combined treatment of TP1-IM + L and anti-PD-L1 had no significant toxic or side effects on mice.

Furthermore, a more aggressive mouse model of abdominal metastasis was established by subcutaneously injecting ID8 cells into the left hips of C57/BL6 mice at 35 days and intraperitoneal injection of ID8 cells at 1 day separately before treatment (Fig. S8). The treatment protocol was the same as the bilateral ID8 subcutaneous tumor mouse model, and bioluminescence imaging *in vivo* was conducted to measure the tumor growth in different groups of mice. Consistent with previous results, the TP1-IM + L + anti-PD-L1 group could significantly inhibit the growth of peritoneal metastases in tumor-bearing mice when compared to that of all other groups. The above results showed that TP1-IM-basedPTT combined with PD-L1 blockade therapy could be an effective strategy to inhibit ID8 tumor metastasis.

### 3.7 The mechanism study of immune response against metastasis *in vivo*

**Fig. 7:**
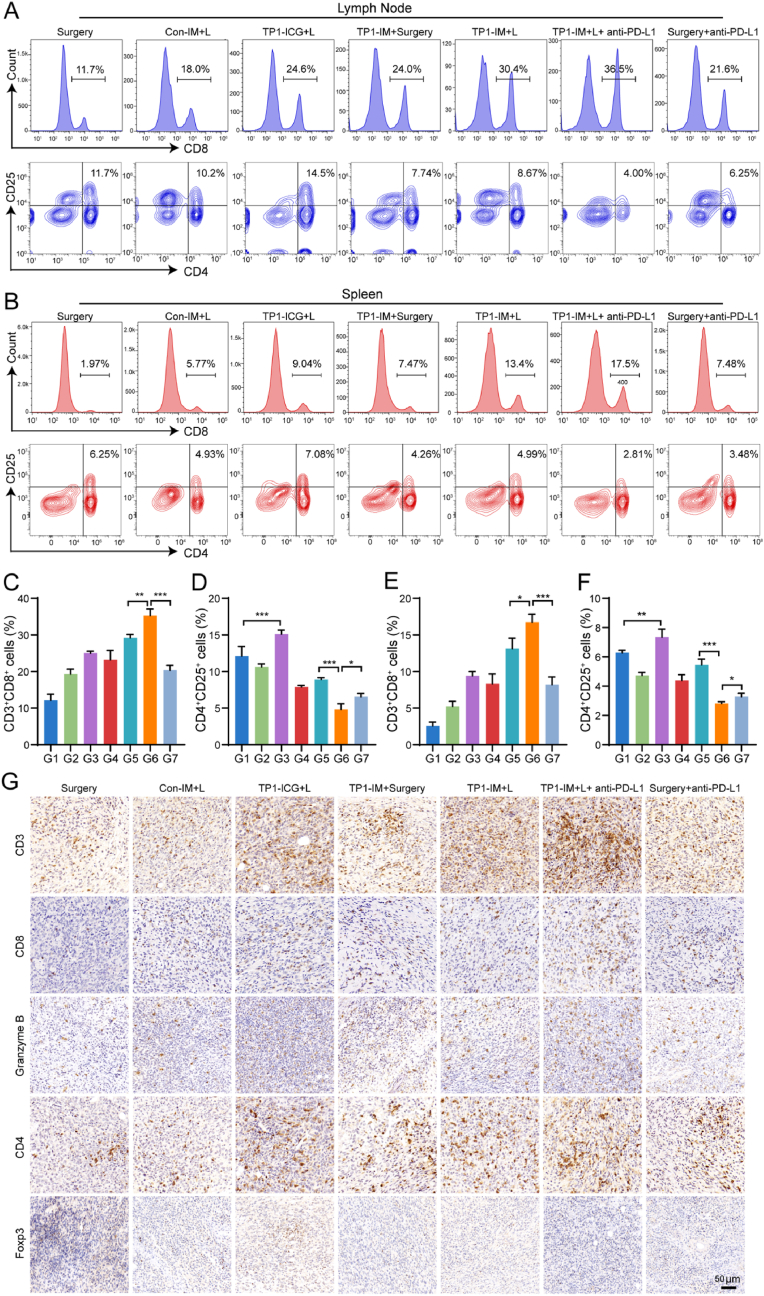
The anti-tumor immune response mediated by TP1-IM-based PTT combined with PD-L1 checkpoint blockade. (A–B) Proportion of cytotoxic T cells (CTLs, CD3^+^CD8^+^) and Treg cells (CD3^+^CD4^+^CD25^+^) in the lymph nodes (A) and spleen (B) on bilateral subcutaneous tumor models after different treatment. (C–F) Analysis of the proportion of CTLs and Treg cells in the lymph nodes (C–D) and spleen (E–F) on bilateral subcutaneous tumor models after different treatment. G1: Surgery; G2: Con-IM + L; G3: TP1-ICG + L; G4: TP1-IM + Surgery; G5: TP1-IM + L; G6: TP1-IM + L + anti-PD-L1; G7: Surgery + anti-PD-L1. +L indicated that the group was treated with NIR irradiation. (G) Representative images of tissue slices of the secondary tumors. The immunoinfiltrated T cells in distal tumor tissue of bilateral tumor models were analyzed by immunohistochemistry assay. Scale bar: 50 μm. ∗: *p* < 0.05, ∗∗: *p* < 0.01, ∗∗∗: *p* < 0.001, ∗∗∗∗: *p* < 0.0001.

To explore the mechanisms of TP1-IM-based PTT plus PD-L1 blockade against tumor metastasis, systemic antitumor immune responses were first analyzed. The TDLNs and spleens of mice in the above groups were collected and further processed into the single-cell suspension and then were analyzed for the proportion of cytotoxic T cells (CTLs, CD3^+^CD8^+^) and Treg cells (CD3^+^CD4^+^CD25^+^) by flow cytometry, and the gating strategy was shown in Fig. S9. As shown in Fig. 7A–B, 7C and 7E, the percentages of CTLs in TDLNs and spleens had certain content of increase in the TP1-ICG + L, TP1-IM and Surgery + anti-PD-L1 monotherapy group when compared with Surgery group, combination therapy such as TP1-IM + L could obviously elevate the CTLs levels, and TP1-IM + L followed PD-L1 blockade were most potent. The cytokine-producing CD8^+^ T cells (Granzyme B, TNF-*α*, and IFN-*γ*) in TDLNs and spleens showed the most significant improvement in the TP1-IM + L with PD-L1 blockade group, compared with TP1-ICG + L, TP1-IM, and Surgery + anti-PD-L1 monotherapy group (Fig. S10). As presented in Fig. 7A–B, 7D, and 7F, compared to the Surgery group, the proportion of Treg cells in the TP1-ICG + L group was upregulated, suggesting the presence of immune suppression after PTT. However, as an immune checkpoint blockade approved by the FDA for cancer treatment, anti-PD-L1 in this experiment could downregulate the proportion of Treg cells in TDLNs and spleens. It was found that the TP1-IM + L + anti-PD-L1 group significantly reduced the proportion of Treg cells in TDLNs and spleens.

Furthermore, tumor tissues in the secondary tumor were isolated and analyzed by immunohistochemistry staining. As shown in Fig. 7G, the infiltration proportion of CD3^+^, CD8^+^, Granzyme B^+^ and CD4^+^ T cells in distant tumor tissues in the TP1-IM + L plus PD-L1 blockade group was significantly increased than that in the single-drug group and the TP1-IM + L group. Notably, compared to the Surgery group, the TP1-ICG + L group increased the proportion of Foxp3^+^ T cells in tumor tissues, while the combination therapy as TP1-IM + L + anti-PD-L1 group could significantly reduce the infiltration ratio of Foxp3^+^ T cells in secondary tumor tissues. The above results indicated that the combination therapy of TP1-IM-based PTT and PD-L1 blockade could effectively elicit the body's anti-tumor immune response by promoting the infiltration of CTLs, reducing the proportion of Treg cells in tumor tissues and producing a systemic anti-tumor immune response.

### 3.8 *In vivo* evaluation of long-term immune memory effect against recurrence

**Fig. 8:**
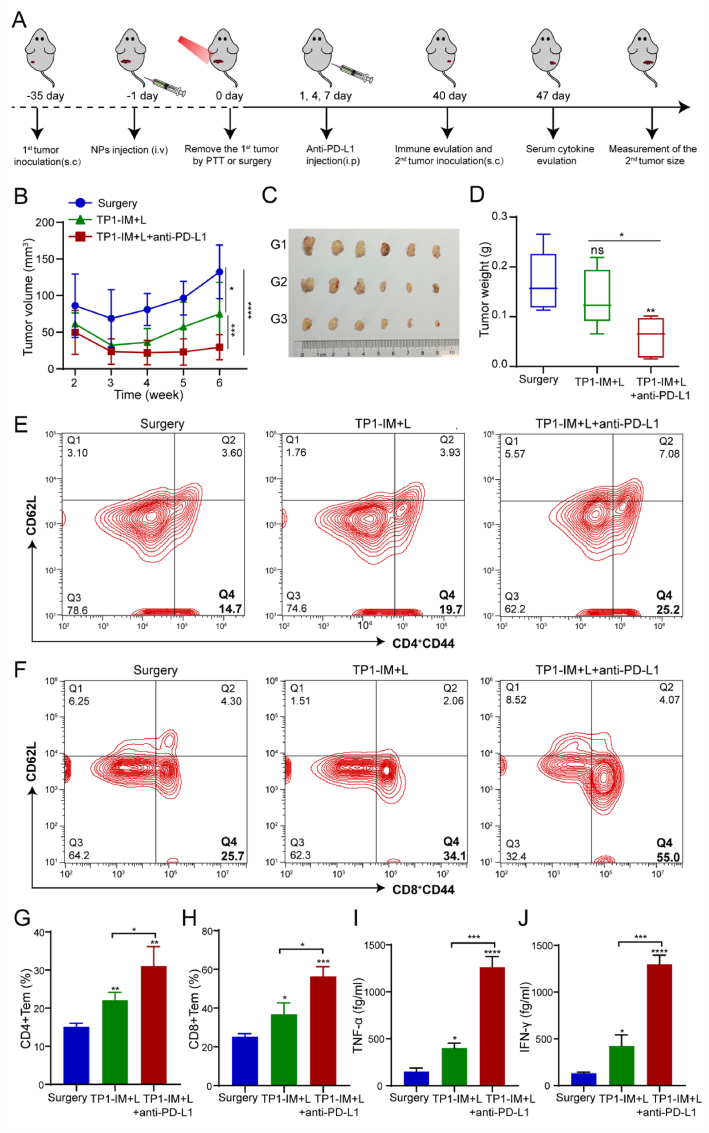
Inhibition of OC recurrence by TP1-IM mediated PTT combined with PD-L1 checkpoint blockade. (A) Schematic illustration of the therapeutic schedule of tumor recurrent mouse model. Mice with ID8 tumors were used in our experiment. (B) Growth curves of the second tumors after different treatment. (C) The image of the secondary tumors after different treatment at the endpoint of observation. G1: Surgery; G2: TP1-IM + L; G3: TP1-IM + L + anti-PD-L1. (D) The secondary tumor weight after indicated treatments was quantitatively analyzed. (E–F) The T_EM_ (CD44^high^CD62^low^) proportion of CD4^+^ T cells and CD8^+^ T cells in spleen of treated mice was detected by flow cytometry on day 40. (G–H) Quantitative analysis of T_EM_ ratio of CD4^+^ T cells and CD8^+^ T cells in spleen of mice in each group. (I–J) The expression levels of TNF-α and IFN-γ in serum of treated mice were detected by CBA on day 47. ∗: *p* < 0.05, ∗∗∗: *p* < 0.001.

The immune system is characterized by its immune memory to prevent diseases, which enables the body to rapidly activate its immune response to eliminate re-invader antigens [57]. In this study, we investigated whether the combination of TP1-IM plus PD-L1 blockade therapy could inhibit tumor recurrence. As shown in Fig. 8A, ID8 cells were inoculated into the left buttock of C57/BL6 mice as the primary tumor site and randomly divided into the three treatment groups: (G1) Surgery; (G2) TP1-IM + L; (G3) TP1-IM + L + anti-PD-L1. G1 received an intravenous injection of PBS, while G2 and G3 received a TP1-IM injection. After 24 h of drug administration, the primary tumor sites of mice in each group were removed through NIR laser irradiation (808 nm, 2 W/cm^2^, 5 min) or surgery on day 0. Anti-PD-L1 antibodies were intraperitoneally injected into the mice in Group 3 on Days 1, 4, and 7. On Day 40, ID8 cells were subcutaneously inoculated into the right buttocks of mice to simulate tumor recurrence, and the second tumor was monitored. As shown in Fig. 8B–C, compared with the Surgery group, both the TP1-IM + L and the TP1-IM + L + anti-PD-L1 groups could inhibit the growth of the second tumor. The TP1-IM + L + anti-PD-L1 group showed a significantly better effect in inhibiting secondary tumor growth than the TP1-IM + L group. At the endpoint of observation, it was found that the tumor weight in the TP1-IM + L + anti-PD-L1 group was the lightest (Fig. 8D). These results suggested that TP1-IM-based PTT combined with PD-L1 blockade could effectively suppress ID8 tumor recurrence through synergistic PTT and immunotherapy.

Furthermore, the mice were sacrificed to assess the immune memory response, and the spleens were collected for further analysis by flow cytometry. The proportions of central memory T cells (T_CM_) and effector memory T cells (T_EM_, CD44^high^CD62L^low^) in the spleen tissues of mice were measured and analyzed. As shown in Fig. 8E–H, compared with the Surgery group, both the TP1-IM + L group and the TP1-IM + L + anti-PD-L1 combination group increased the proportion of CD4^+^ T_EM_ and CD8^+^ T_EM_ cells. However, the proportion of T_EM_ was much higher in the TP1-IM + L + anti-PD-L1 group. The mouse serum was isolated, and the cytokines TNF-α and IFN-γ levels were measured by flow cytometry on day 47. As shown in Fig. 8I–J, the proinflammatory cytokines, such as TNF-α and IFN-γ, were significantly increased in the TP1-IM + L + anti-PD-L1 group. These results indicated that the combination treatment of TP1-IM micelles and anti-PD-L1 blockade could obviously stimulate the body to produce an effective antitumor immune memory response to prevent tumor recurrence.

## 4 Conclusion

In summary, our study successfully synthesized a type of micelles with multifunction. The obtained TP1-IM micelles could actively target OC cells, present good stability and retain the photothermal effect of ICG. Under NIR laser irradiation *in vitro*, the TP1-IM micelles could effectively destroy OC cells, induce ICD, and stimulate DCs maturation. *In vivo*, the photothermal therapy and immunotherapy mediated by TP1-IM micelles could promote the maturation of DCs, effectively inhibit the growth of OC and prolong the survival of tumor-bearing mice. Combined with PD-L1 checkpoint blockade, it could obviously promote the infiltration of cytotoxic T cells at the tumor site, relieve an immunosuppressive environment and induce an anti-tumor immune response. The combined therapy could significantly inhibit the metastasis and recurrence of OC, providing a new option for the individualized treatment of OC in the future.

## CRediT authorship contribution statement

**Ling Wang:** Writing – review & editing, Writing – original draft, Validation, Project administration, Methodology, Investigation, Funding acquisition, Formal analysis. **Jie Li:** Writing – review & editing, Validation, Project administration, Methodology, Investigation. **Danya Zhang:** Writing – review & editing, Writing – original draft, Project administration, Methodology, Investigation. **Songwei Tan:** Supervision, Project administration, Methodology, Investigation. **Guiying Jiang:** Project administration, Methodology, Investigation. **Xueqian Wang:** Project administration, Methodology, Investigation. **Fei Li:** Supervision, Project administration, Methodology, Investigation, Funding acquisition. **Ying Zhou:** Writing – review & editing, Visualization, Project administration, Methodology, Investigation. **Pingbo Chen:** Visualization, Project administration, Methodology, Investigation, Conceptualization. **Rui Wei:** Writing – review & editing, Writing – original draft, Visualization, Validation, Supervision, Investigation, Funding acquisition, Conceptualization. **Ling Xi:** Writing – review & editing, Writing – original draft, Visualization, Supervision, Project administration, Investigation, Funding acquisition, Formal analysis, Data curation, Conceptualization.

## Ethics approval

All the animal experiments were approved by the Experimental Animal Welfare Ethics Committee of Tongji Hospital, Affiliated with Huazhong University of Science and Technology. (TJH-202005003).

## Funding

This work was supported by the 10.13039/501100001809National Natural Science Foundation of China (82002764, 82172717, 82102884, 82272628, 82303756).

## Declaration of competing interest

The authors declare no conflict of interest.

## Data Availability

Data will be made available on request.
