# Supplementary material for: TMTP1-modified polymeric micelles for the inhibition of ovarian cancer metastasis and recurrence through enhanced photothermal-immunotherapy
Source: Mater Today Bio. 2025 May 4;32:101825. doi: 10.1016/j.mtbio.2025.101825 (PMC12140946; doi:10.1016/j.mtbio.2025.101825)
Supplement: Multimedia component 1 [file mmc1.docx]

Supporting Information

**TMTP1-Modified Polymeric Micelles for the Inhibition of Ovarian Cancer Metastasis and Recurrence through Enhanced Photothermal-Immunotherapy**

*Ling Wang, Jie Li, Danya Zhang, Songwei Tan, Guiying Jiang, Xueqian Wang, Fei Li, Ying Zhou, Pingbo Chen, Rui Wei*, Ling Xi**

Table S1: Optimization of the preparation conditions for TP1-IM micelles

| PBAE/ICG  (mass ratio) | ICG encapsulation efficiency (%) | Hydrated particle size (nm) | PDI |
| --- | --- | --- | --- |
| 0 | 20.91 ± 1.66 | 141.00 ± 2.31 | 0.09 ± 0.08 |
| 1:1 | 45.65 ± 0.88 | 157.53 ± 2.28 | 0.07 ± 0.01 |
| 2:1 | 78.57 ± 5.41 | 163.90 ± 2.20 | 0.10 ± 0.08 |


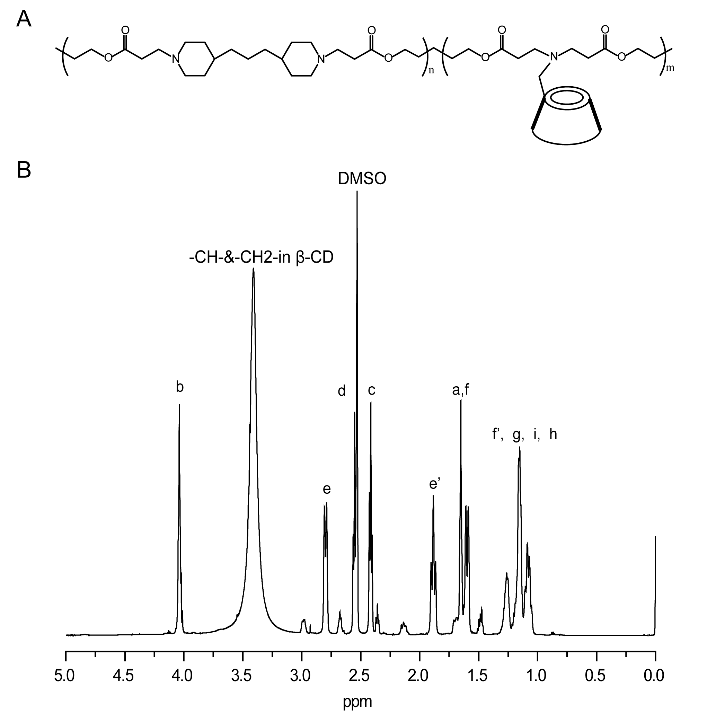


Figure S1：(A) Chemical structural formula of PBAE；(B) The structure of PBAE was confirmed by proton nuclear magnetic resonance.

Table S2: The effects of PBAE on MPLA encapsulation efficiency of TP1-IM micelles

| PBAE | MPLA encapsulation efficiency (%) |
| --- | --- |
| + | 90.33 ± 0.37 |
| - | 61.29 ± 0.30 |

Table S3: The hydrated particle size, zeta potential and ICG encapsulation efficiency of nanomicelles

| Nanomicelles | Hydrated particle size (nm) | PDI | Zeta potential (mV) | ICG encapsulation efficiency (%) |
| --- | --- | --- | --- | --- |
| Con-IM | 157.30 ± 4.80 | 0.06 ± 0.05 | -18.99 ± 2.47 | 78.38 ± 4.55 |
| TP1-ICG | 167.20 ± 8.20 | 0.12 ± 0.05 | -13.25 ± 1.34 | 79.06 ± 5.55 |
| TP1-MP | 167.50 ± 8.70 | 0.06 ± 0.02 | -23.97 ± 2.41 | / |
| TP1-IM | 165.50 ± 8.20 | 0.07 ± 0.05 | -9.93 ± 1.52 | 78.57 ± 5.41 |


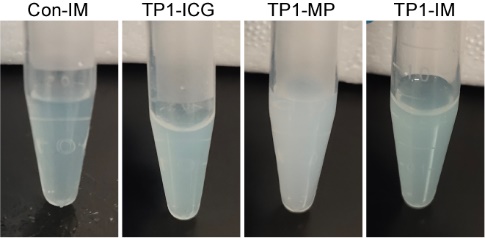


Figure S2：The appearance of Con-IM, TP1-ICG, TP1-MP and TP1-IM micelles.


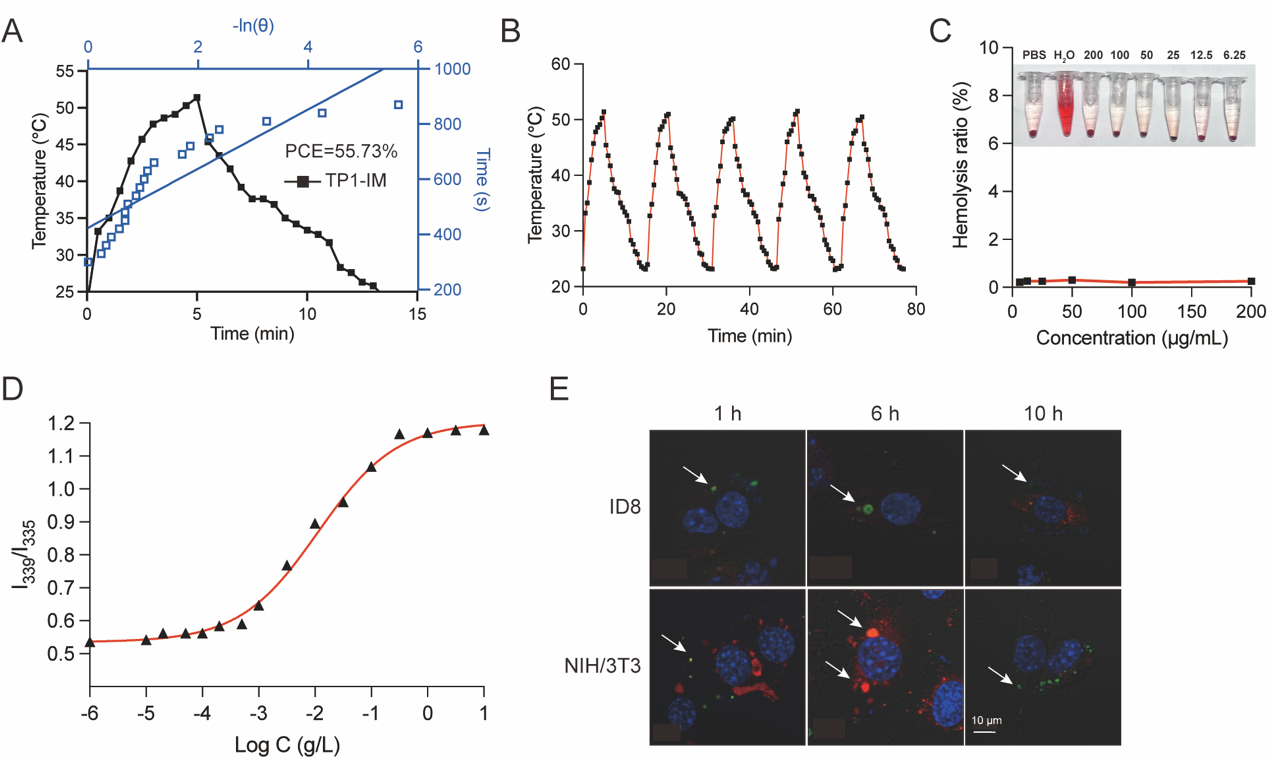


Figure S3: (A) The photothermal conversion efficiency of TP1-IM micelles. (B) The heating and cooling curves of TP1-IM under 808 nm irradiation over five circles. (C) The hemolysis ratio of TP1-IM at different concentrations (200, 100, 50, 25, 12.5, and 6.25 μg/mL), the H_2_O served as the positive control, and PBS as the negative control. (D) The critical micelle concentration of TP1-IM micelles; (E) The intracellular localization of TP1-CM micelles *in vitro*. Confocal images of ID8 and 3T3 cells incubated with TP1-CM micelles for 1 h, 6 h, 10 h, and stained with Lyso-Tracker Red. Green fluorescence: Coumarin 6, TP1-CM; Red fluorescence: Lyso-Tracker Red dye, Lysosome. Scale bar: 10 µm.


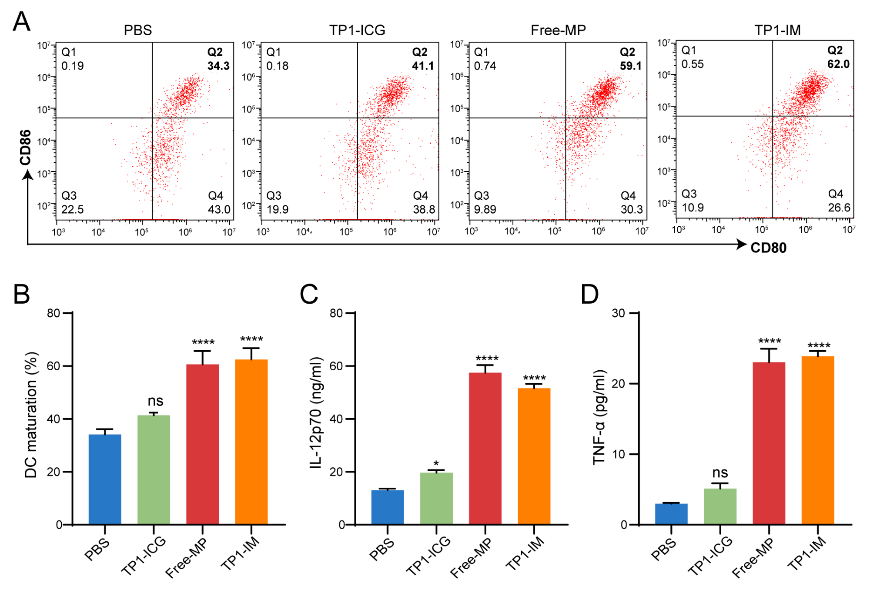


Figure S4: Immune activation of TP1-IM micelles *in vitro*. (A) The proportion of DCs maturation (CD11c^+^CD80^+^CD86^+^) of Free MPLA (Free-MP), TP1-ICG and TP1-IM micelles containing the same MPLA (1µg/ml) was determined by flow cytometer after incubation with BMDCs for 12 hours, respectively. The BMDCs group treated with PBS under the same conditions was used as the negative control. (B) Quantitative analysis of DCs maturation stimulated by PBS, TP1-ICG, Free-MP and TP1-IM micelles. (C-D) The expression levels of IL-12p70 and TNF-α in the supernatant of DCs treated with PBS, TP1-ICG, Free-MP and TP1-IM micelles. n.s: not significant, *: *p* < 0.05, ****: *p* < 0.0001.


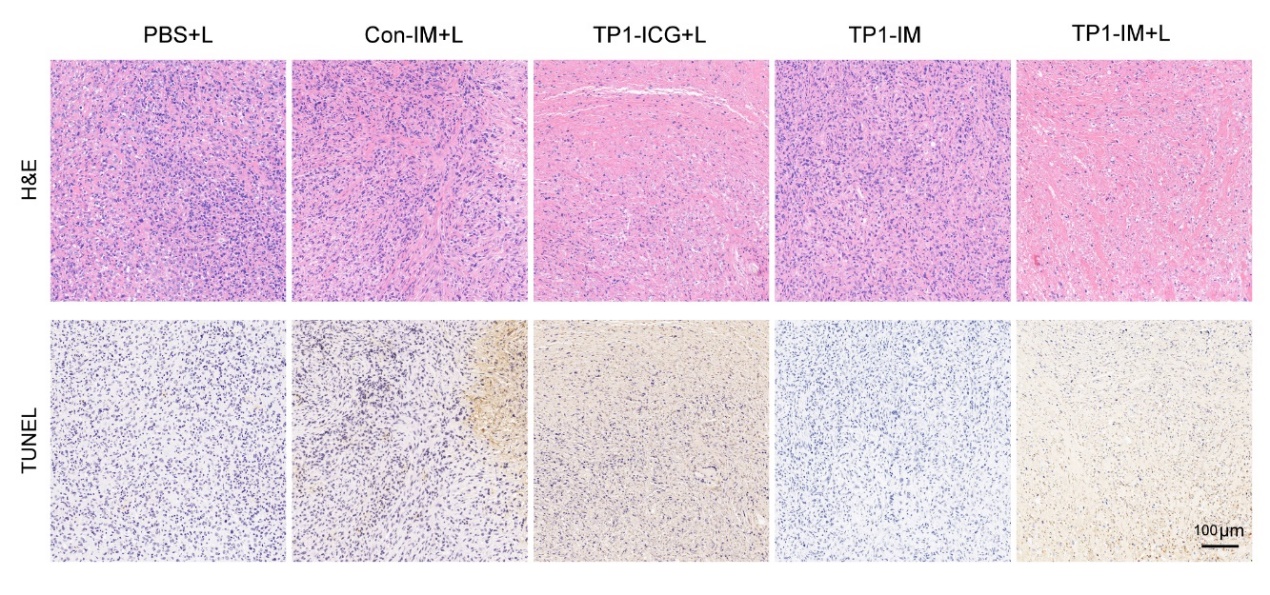


Figure S5: Representative H&E and TUNEL staining images of tumor tissues in each group after PTT. Scale bar: 100 μm.


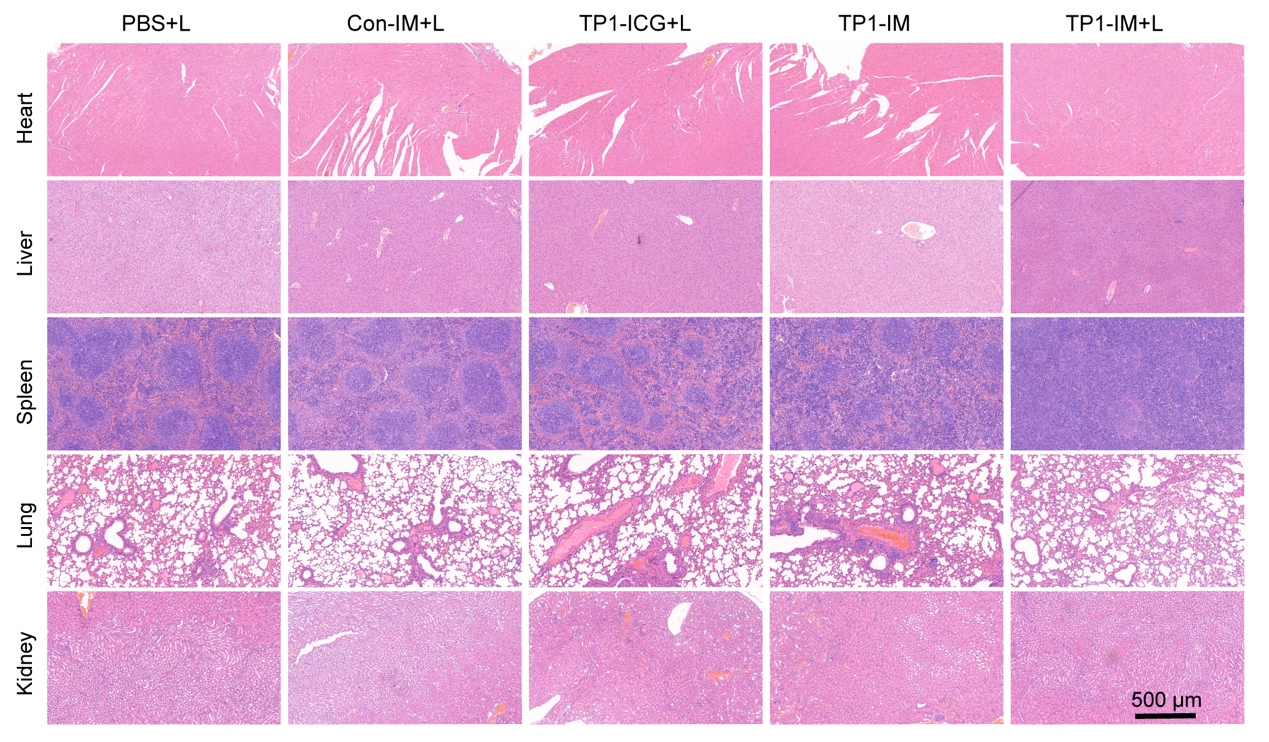


Figure S6: The major organs (heart, liver, spleen, lung, kidney) were separated for HE staining after different treatments. Scale bar: 500 μm.


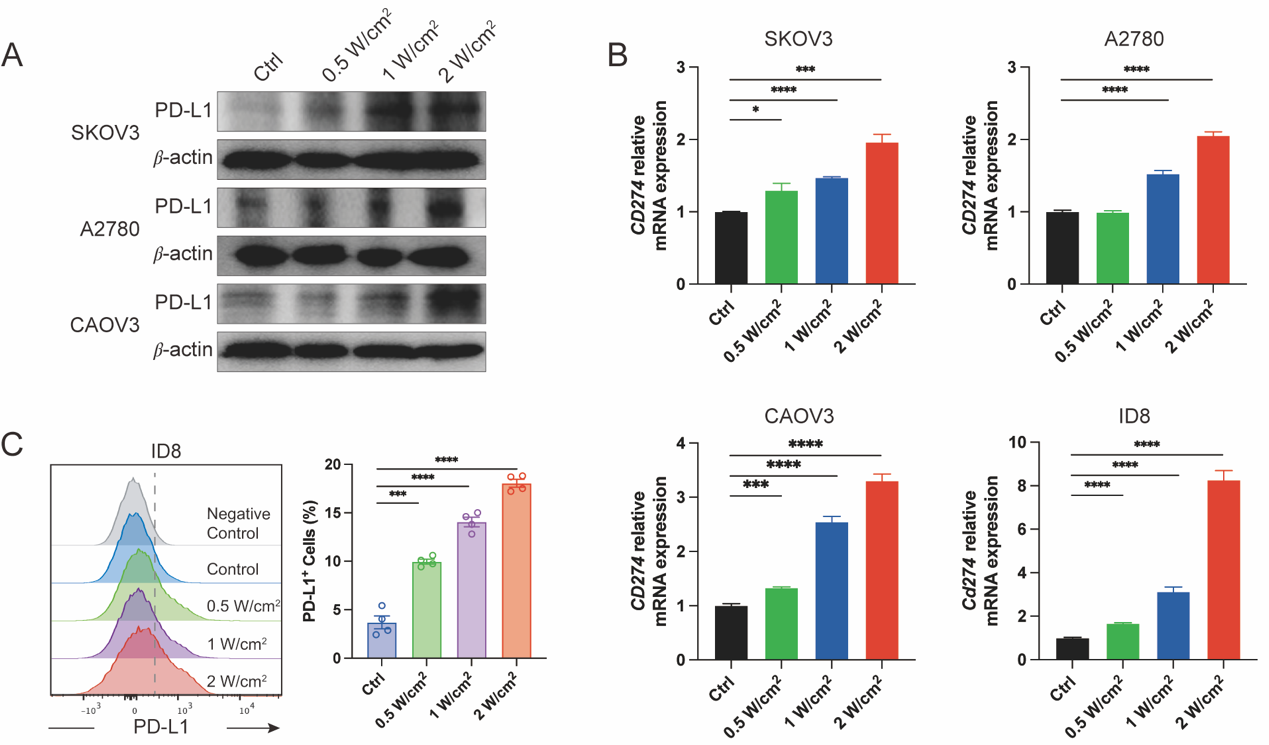


Figure S7: The PD-L1 expression level on ovarian cancer cells after PTT was detected by western blot (A), RT-qPCR (B), and flow cytometry (C). *：*p* < 0.05, ***: *p* < 0.001, ****: *p* < 0.0001.

Table S4: Primer sequences used in quantitative RT-PCR.

| Gene name | Primer sequence |
| --- | --- |
| CD274 | Forward：5’ GCTGCACTAATTGTCTATTGGGA 3’  Reverse：5’ AATTCGCTTGTAGTCGGCACC 3’ |
| GADPH | Forward：5’ GGAGCGAGATCCCTCCAAAAT 3’  Reverse：5’ GGAGCGAGATCCCTCCAAAAT 3’ |
| Cd274 | Forward：5’ GCTCCAAAGGACTTGTACGTG 3’  Reverse：5’ TGATCTGAAGGGCAGCATTTC 3’ |
| Gadph | Forward：5’ AGGTCGGTGTGAACGGATTTG 3’  Reverse：5’ TGTAGACCATGTAGTTGAGGTCA 3’ |

Figure S8:
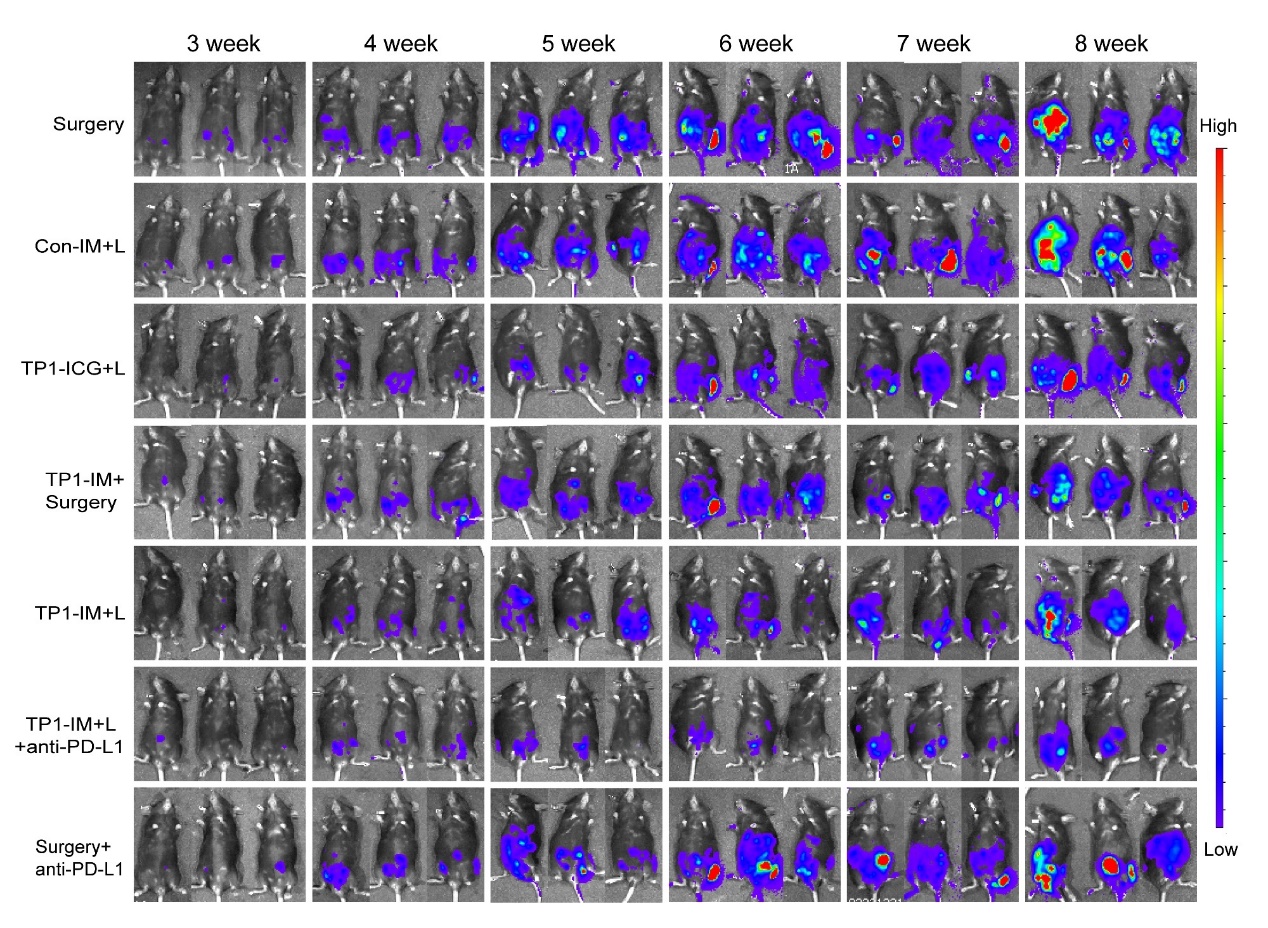
TP1-IM micelles-based PTT combined with PD-L1 checkpoint blockade inhibited the growth of peritoneal metastases.


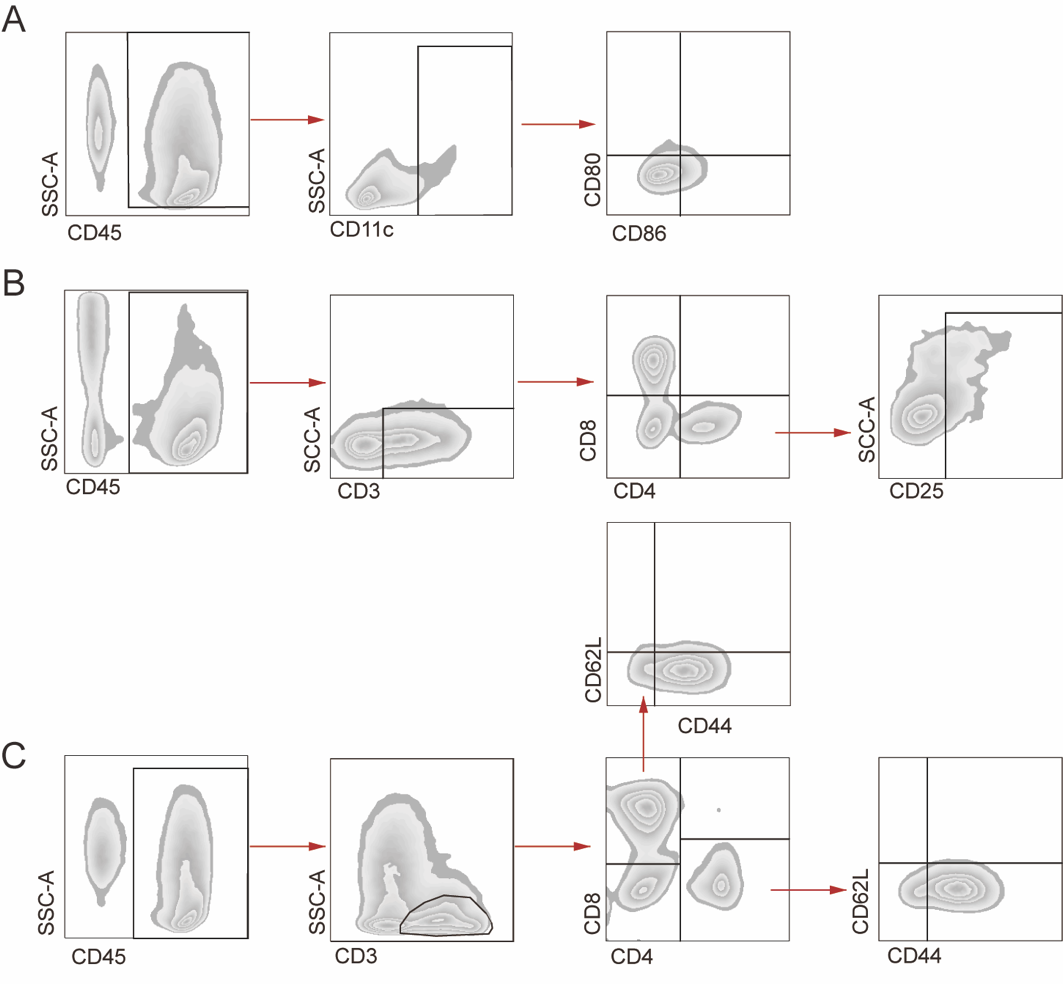


Figure S9: The gating strategies scheme. (A) Gating strategies for dendritic cells. (B) Gating strategy for CD8^+^ T cells and CD4^+^CD25^+^ T cells. (C) Gating strategy for CD4^+^CD44^+^CD62L^+^ T cells and CD8^+^CD44^+^CD62L^+^ T cells.


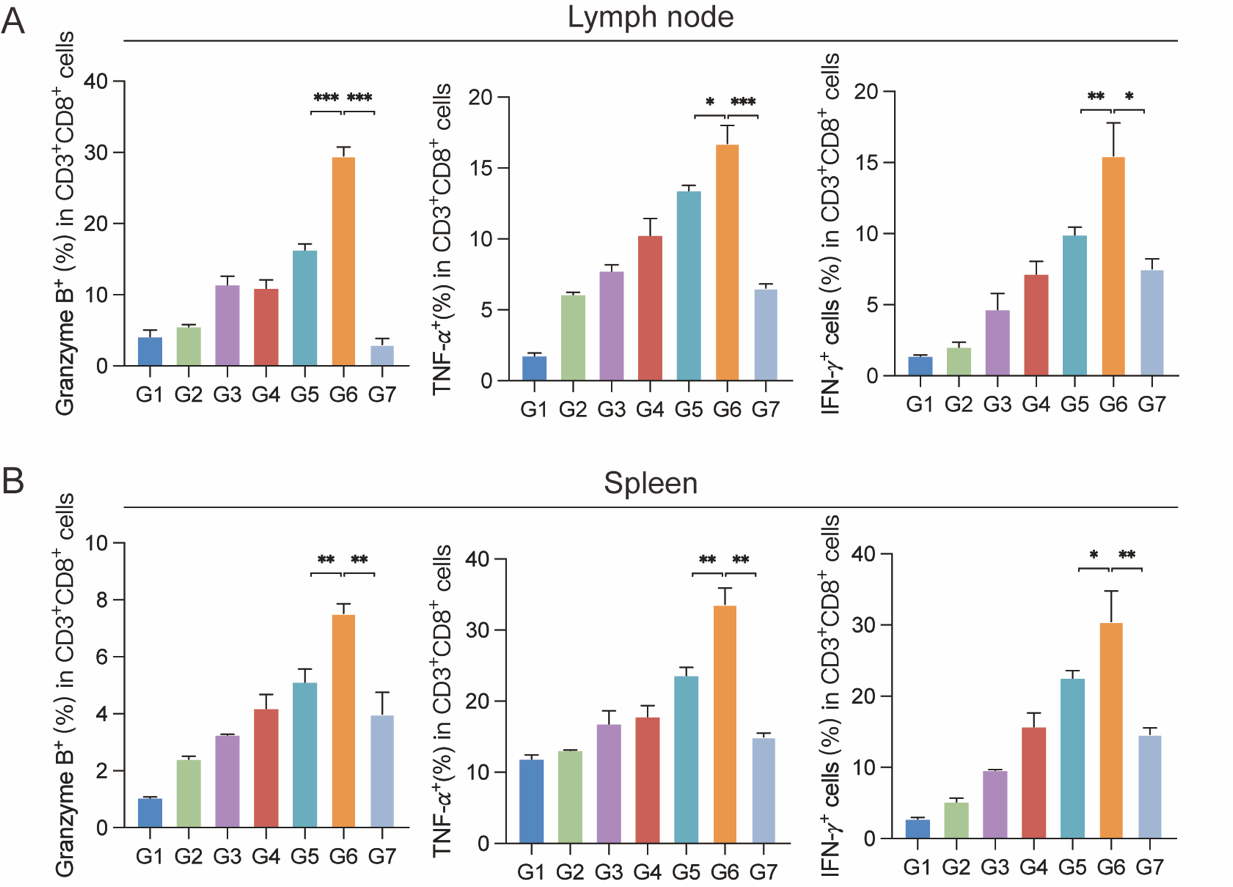


Figure S10: The cytokine producing CD8^+^ T cells (Granzyme B, TNF-𝛼, and IFN-𝛾) from TDLNs (A) and spleen (B) on bilateral subcutaneous tumor models after different treatments were analyzed by flow cytometry. G1: Surgery; G2: Con-IM+L; G3: TP1-ICG+L; G4: TP1-IM+Surgery; G5: TP1-IM+L; G6: TP1-IM+L+anti-PD-L1; G7: Surgery+anti-PD-L1. +L indicated that the group was treated with NIR irradiation. *：*p* < 0.05，**: *p* < 0.01, ***: *p* < 0.001.
